# Impact of clonal hematopoiesis on cardiovascular outcomes in cancer patients of the UK Biobank

**DOI:** 10.1016/j.esmoop.2025.105539

**Published:** 2025-08-07

**Authors:** M. Sun, L. Busque, J. Sandoval, L.-P. Lemieux Perreault, A. Barhdadi, M.-C. Cyr, J.-C. Tardif, M.-P. Dubé

**Affiliations:** 1 Montreal Heart Institute, Montreal, Canada; 2 Université de Montréal Beaulieu-Saucier Pharmacogenomics Centre, Montreal, Canada; 3 Department of Medicine, Faculty of Medicine, Université de Montréal, Montreal, Canada; 4 Hôpital Maisonneuve-Rosemont, Montreal, Canada

**Keywords:** clonal hematopoiesis, CHIP, mosaic chromosomal alterations, cardiovascular mortality, UK Biobank, cancer

## Abstract

**Background:**

Clonal hematopoiesis of indeterminate potential (CHIP) and mosaic chromosomal alterations (mCAs) have been linked to increased risks of cardiovascular disease (CVD) and mortality. CHIP and mCAs may also then contribute to CVD in cancer patients. Our objective was to investigate the prevalence of CHIP mutations and mCAs in cancer patients, their co-occurrence, and the associated CVD outcomes across different cancer types.

**Patients and methods:**

We carried out a case-control analysis of CHIP and mCA on the risks of CVD-related outcomes using the UK Biobank. Somatic CHIP mutations were identified from whole-exome sequencing and mCAs from genotyping data among patients diagnosed with cancers. Logistic regression and Cox proportional hazards models were used to assess the associations between CHIP mutations, mCAs, and CVD outcomes, and overall mortality.

**Results:**

Overall, 2701 patients (5.5%) harbored CHIP mutations. Increasing age, current smoking, and chemotherapy exposure were associated with higher odds of CHIP mutations and mCAs. Co-occurrence of CHIP and mCAs was observed in 695 patients (25.7% of those with CHIP mutations). Loss of the Y chromosome (LOY) was inversely correlated with CHIP mutations among men [odds ratio (OR) 0.65, 95% confidence interval (CI) 0.57-0.74, *P* < 0.001] whereas loss of the X chromosome (LOX) was positively correlated with CHIP mutations among women (OR 1.24, 95% CI 1.03-1.49, *P* = 0.03). CHIP mutations were associated with an increased risk of incident CVD [hazard ratio (HR) 1.07, 95% CI 1.02-1.13, *P* = 0.004] and overall mortality (HR 1.31, 95% CI 1.22-1.40, *P* < 0.001). Notably, there was no synergistic impact of CHIP mutations co-occurring with mCAs (LOY/LOX) on considered outcomes.

**Conclusions:**

CHIP mutations and mCAs are prevalent in cancer patients and are associated with significant increases in cardiovascular risk and mortality, with variations across cancer types. These findings underscore the importance of considering clonal hematopoiesis in the clinical management of cancer patients to mitigate cardiovascular risks.

## Highlights

• CHIP prevalence in cancer is 5.5%.
• CHIP boosts CVD and overall death.
• There is no CHIP–mCA synergy.

## Introduction

Clonal hematopoiesis (CH) in hematopoietic stem and progenitor cells was first characterized in aging women with X chromosome inactivation skewing.^1^ Whole-exome sequencing (WES) of large cohorts revealed that CH, later defined as clonal hematopoiesis of indeterminate potential (CHIP)^2^, ^3^, ^4^—referring to CH with a cancer-associated somatic mutation detected in the blood at a variant allele frequency (VAF) of ≥2%—has been found to increase with age and is linked to a higher risk of hematologic malignancy.^3^^,^^5^ CHIP mutations also correlate with a greater risk of all-cause mortality, largely driven by cardiovascular-related causes,^6^ likely due to a shared inflammatory pathophysiology.^7^^,^^8^

Given the connection between CHIP and cardiovascular events in the general population, it is imperative to understand whether this association holds or is amplified in cancer patients. In particular, patients diagnosed with cancer have worse cardiovascular health,^9^^,^^10^ which may also be exacerbated by cancer-related treatments.^11^^,^^12^ The reliance of markers that can better identify patients at higher risk of cardiovascular problems can improve prediction and prevention strategies, including enhanced cardiovascular surveillance and pharmacological interventions. We have previously shown that mosaic chromosomal alterations (mCA)—acquired structural alterations in hematopoietic cells indicative of CH—are also associated with increased cardiovascular disease (CVD) risk in patients with cancer.^13^ Therefore, exploring the connection between CHIP and mCA, and their combined impact on cardiovascular risks in cancer patients, is crucial. This investigation can enhance our comprehensive understanding of how CH influences cardiovascular outcomes.

This study sought to assess the effect of common CHIP mutations on cardiovascular health and survival outcomes in a patient cohort comprising exclusively of individuals diagnosed with cancer, and to examine the clinical significance of co-occurring CHIP and mCAs on these outcomes.

## Methods

### Data source

The UK Biobank is a prospective cohort study with genetic and phenotypic data from ∼500 000 participants from across the UK aged between 40 and 60 years at recruitment.^14^ Baseline assessments included DNA sample collection. Of 469 918 participants with WES (as of 7 March 2023), we focused on those with cancer types known to be at increased cardiovascular mortality risk,^9^^,^^13^ by using diagnoses (ICD-10) from linked cancer registry data (Supplementary Table S1, available at https://doi.org/10.1016/j.esmoop.2025.105539). We excluded participants with hematologic cancers diagnosed within ±6 months of study entry, individuals without genotypic–phenotypic sex concordance, and one of each pair of first- and second-degree relatives at random, leaving 49 149 participants for analyses.

### CHIP calling

We called somatic mutations using Mutect2, focusing on 11 well-defined and recurrent CHIP-associated genes: *ASXL1*, *CBL*, *DNMT3A*, *GNAS*, *GNB1*, *JAK2*, *PPM1D*, *SF3B1*, *SRSF2*, *TET2*, and *TP53.* Variants from gnomAD v2 were used as a reference for germline allele frequency, with the Genome Analysis Toolkit (GATK) panel of normals from the 1000 Genomes Project (1KGP). Unfiltered variants were annotated with Annovar and flagged as CHIP (or whitelisted) if they match a prespecified list.^6^^,^^15^ We applied sequencing depth filters [read depth (DP) ≥20; allele depth (AD) ≥5, forward strand read 1 and reverse strand read 2 (F1R2) and forward strand read 2 and reverse strand read 1 (F2R1) read pair depth ≥1] and removed sites within homopolymer runs (a sequence of five identical bases) if AD <10 or VAF <0.08. Artifacts reported by Busque et al.^8^ were removed, including the TP53:NM_000546:p.P72R, ASXL1:NM_015338:P815L, and ASXL1 p.G646Wfs∗12 variants with a VAF <0.1). Additionally, missense mutations in *CBL*, *TET2*, *DNMT3A*, and *TP53* that were inconsistent with somatic mutations (i.e. with a *P* value ≥0.01 in a binomial test of VAF = 0.5) were excluded. TET2:p.H1904R, TET2:p.I1873T, and TET2:p.T1884A were exempt from binomial tests as Vlasschaert et al.^15^ suggested that these are likely CHIP variants. Variants present in >20 individuals were tested for association with age and *TERT* variant rs7705526, and only included if *P* ≤ 0.1. Finally, artifacts reported by Vlasschaert et al.^15^ based on the analysis of 454 787 UK Biobank participants were also removed.

### mCA calling

As previously described,^16^^,^^17^ allele-specific single nucleotide polymorphism (SNP)-array intensity data obtained by genotyping blood-derived DNA from UK Biobank participants were used to call mCAs. mCAs were determined from genotype intensities log_2_R ratio (LRR) and B-allele frequency (BAF) values, which were used to estimate the total and relative allelic intensities, respectively. Rephasing was conducted using Eagle2^18^ and mCA calling leveraged long-range phase information searching for allelic imbalances between maternal and paternal allelic fractions across contiguous genomic segments. For the purpose of our study, mCA calls were obtained from dataset return #3094 from the UK Biobank application #19808,^16^^,^^18^ and categorized as any mCA, LOY, LOX, and expanded mCAs (≥10% of cell fraction).

### Phenotypic definitions

Our primary endpoints included incident CVD, incident coronary artery disease (CAD), time to death from CVD causes, time to death from CAD causes, and time to death from any causes. Incident CVD and CAD were derived using previous definitions.^19^ Cause of death was based on ICD-10 codes for primary cause of death per death register records. For each endpoint, if patients had a cancer diagnosis before baseline, the time to event (in years) was calculated from assessment visit date. If cancer diagnosis occurred after baseline, time to death was calculated from cancer diagnosis date. To overcome time bias incurred due to prevalent cancer diagnoses, we calculated the number of days between prevalent cancer diagnosis date and study recruitment, setting it as 0 if the cancer diagnosis occurred after baseline. For individuals who were not deceased or without incident cardiovascular events, follow-up ended on the last registered death date (Supplementary Figure S1, available at https://doi.org/10.1016/j.esmoop.2025.105539).

### Statistical analysis

In our primary analysis, we examined endpoints using Cox proportional hazards regression models, evaluating the impact of CHIP (any versus none) across all cancer patients, adjusting for age, sex, smoking status (never smokers; individuals who have never smoked tobacco, former smokers: individuals who have smoked tobacco in the past but do nott currently smoke, current smoker: individuals who currently smoke tobacco), chemotherapy, radiotherapy, prevalent CVD, the interval between recruitment and cancer diagnosis, and the first 10 principal components of genetic ancestry.

We further analyzed the influence of CHIP mutations with mCAs on our primary outcomes, assessing CHIP-by-mCA interaction effects. Additive and multiplicative interactions between CHIP and mCA on CV risk were evaluated using the relative excess risk due to interaction (RERI), the attributable proportion (AP), and the synergy index (SI).^20^^,^^21^ The RERI quantifies the excess risk due to the interaction above the sum of the individual risks. The AP represents the proportion of the risk that is attributed to the interaction between CHIP and mCA. SI measures the multiplicative interaction between CHIP and mCA.

We also evaluated the expanded CHIP mutations (VAF ≥10%) and mCAs (>10% cell fraction).^15^^,^^16^ We repeated analyses for each cancer type, including a CHIP status-by-cancer type interaction term, to determine if the effects of CHIP on the endpoints varied depending on the type of cancer diagnosed. All analyses were carried out using Jupyter notebook (version 5.0), developed by Project Jupyter (Berkeley, CA). The analyses were conducted on the DNAnexus Platform, provided by DNAnexus, Inc (Mountain View, CA), with the PYTHON_R feature (Python 3.6.5 libraries and R 4.1.3 libraries).^22^ All analytical and summary reports were produced with gtsummary (version 1.6.1).^23^

### Ethics statement

The study was approved by the Montreal Heart Institute research ethics committee and complies with the Declaration of Helsinki.

## Results

### Baseline descriptives of CH

Of 49 149 patients diagnosed with cancer, 2701 harbored somatic CHIP mutations (5.5%, Table 1). Of those, 2279 (84.4%), 233 (8.6%), and 189 (7.0%) had 1, 2, and ≥3 CHIP mutations. The most common mutations were *DNMT3A* (*n* = 1407, 52.1% of all carriers of CHIP mutations), *TET2* (*n* = 606, 22.4%), and *ASXL1* (*n* = 370, 13.7%, Supplementary Figure S2, available at https://doi.org/10.1016/j.esmoop.2025.105539). Overall, 10 157 individuals carried at least one mCA (20.7%, Table 1). Of those, 6534 were LOY (13.2%) and 1652 were LOX (3.4%). Expanded mCAs were detected in 19% of patients with mCAs.

**Table 1:** Descriptive characteristics of patients diagnosed with cancers susceptible to cardiovascular-related health issues, stratified according to CHIP status, UK Biobank (*n* = 49 159)

Characteristic	Overall, *n* (%) *N* = 49 159	No CHIP, *n* (%) *N* = 46 458	CHIP, *n* (%) *N* = 2701	OR^a^	95% CI^a^	*P*
Age at baseline, years				1.080	1.072-1.088	<0.001
Mean (SD)	60 (7)	60 (7)	63 (5)			
Median (IQR)	62 (57-65)	62 (56-65)	64 (61-67)			
Range	40-71	40-71	41-70			
Sex						
Female	26 623 (54.2)	25 312 (54.5)	1311 (48.5)	Ref.	—	
Male	22 536 (45.8)	21 146 (45.5)	1390 (51.5)	1.080	0.998-1.168	0.057
Smoking status						
Current smoker	5513 (11.3)	5144 (11.1)	369 (13.8)	Ref.	—	
Never smoker	23 968 (49.1)	22 817 (49.4)	1151 (43.0)	0.684	0.606-0.774	<0.001
Previous smoker	19 381 (39.7)	18 226 (39.5)	1155 (43.2)	0.764	0.677-0.865	<0.001
Unknown	297	271	26	—	—	
Prevalent CVD	11 126 (22.6)	10 410 (22.4)	716 (26.5)	1.046	0.956-1.144	0.321
Chemotherapy	11 477 (23.3)	10 783 (23.2)	694 (25.7)	1.258	1.149-1.376	<0.001
Radiotherapy	3086 (6.3)	2917 (6.3)	169 (6.3)	0.990	0.840-1.160	0.906
Any mCA	10 157 (20.7)	9462 (20.4)	695 (25.7)	1.056	0.960-1.160	0.263
LOY	6534 (13.2)	6193 (13.3)	341 (12.6)	0.652	0.574-0.739	<0.001
LOX	1652 (3.4)	1533 (3.3)	119 (4.4)	1.242	1.016-1.505	0.030
Expanded mCA	1946 (4.0)	1775 (3.8)	171 (6.3)	1.554	1.311-1.830	<0.001
Expanded CHIP	1713 (3.5)	—	1713 (63.4)	—	—	

Expanded CHIP means variant allele frequency (VAF) ≥10%; expanded mCA means cell fraction >10%.

CHIP, clonal hematopoiesis of indeterminate potential; CI, confidence interval; CVD, cardiovascular disease; IQR, interquartile range; LOX, loss of X chromosome; LOY, loss of Y chromosome; mCA, mosaic chromosomal alterations; OR, odds ratio; Ref., referent category; SD, standard deviation.

a Adjusted for age at baseline and sex (except for mosaic loss of the Y chromosome and the mosaic loss of the X chromosome).

**Figure 1:**
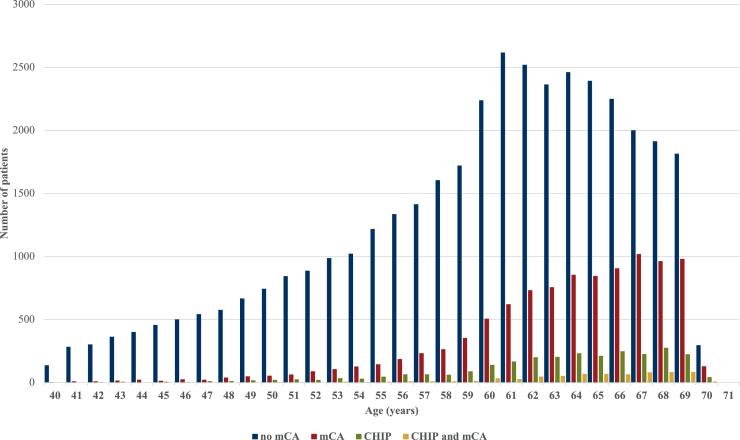
**Prevalence of CHIP and mCA, and both by age in individuals diagnosed with cancer within the UK Biobank.** Bar chart shows the full age distribution of all participants in this cohort at baseline (*n* = 49 519). CHIP, clonal hematopoiesis of indeterminate potential; mCA, mosaic chromosomal alterations.

Increasing age was associated with higher odds of CHIP mutations [odds ratio (OR) 1.08, 95% confidence interval (CI) 1.07-1.09, *P* < 0.001, Table 1, Figure 1], and mCAs (OR 1.10, 95% CI 1.090-1.099, *P* < 0.001, Supplementary Table S2, available at https://doi.org/10.1016/j.esmoop.2025.105539). Compared with current smokers, noncurrent smokers had lower odds of having CHIP mutations (never smokers OR 0.68, 95% CI 0.61-0.77, *P* < 0.001; previous smokers OR 0.76, 95% CI 0.68-0.87, *P* < 0.001, Table 1) or mCAs (Supplementary Table S2, available at https://doi.org/10.1016/j.esmoop.2025.105539). Chemotherapy was associated with greater occurrence of CHIP mutations (OR 1.26, 95% CI 1.15-1.38, *P* < 0.001). The proportion of patients with CHIP mutations in *PPM1D* (5.5% versus 3.1%, *P* = 0.007), *TP53* (3.8% versus 1.5%, *P* < 0.001), and *SF3B1* (1.9% versus 0.8%, *P* = 0.02) was significantly higher in patients treated with chemotherapy than those without (Supplementary Figure S3, available at https://doi.org/10.1016/j.esmoop.2025.105539).

### CHIP and mCA co-occurrence

Overall, 695 patients had CHIP mutations co-occurring with mCAs (25.7% of all CHIP mutations, 1.5% overall, Table 1). mCAs appeared earlier than CHIP mutations (Figure 1). Overall, carriers of mCAs were not at higher odds of co-occurring CHIP mutations, but those with expanded mCAs (cell fraction >10%, representing 19.2% of all mCAs) had significantly higher odds of CHIP mutations (OR 1.55, 95% CI 1.31-1.83, *P* < 0.001, Table 1). LOY was inversely correlated with CHIP mutations (OR 0.65, 95% CI 0.57-0.74, *P* < 0.001), whereas LOX was positively associated with CHIP mutations (OR 1.24, 95% CI 1.31-1.83, *P* = 0.03). Individuals with *TET2* mutations had higher odds of having mCAs (OR 1.24, 95% CI 1.03-1.49, *P* = 0.025, Supplementary Table S2, available at https://doi.org/10.1016/j.esmoop.2025.105539).

### CH per cancer type

Patients diagnosed with lung cancer had higher odds of CHIP mutations (OR 1.57, 95% CI 1.40-1.76, *P* < 0.001, Supplementary Table S3, Figures S4 and S5, available at https://doi.org/10.1016/j.esmoop.2025.105539) and mCAs (OR 1.30, 95% CI 1.20-1.40, *P* < 0.001, Supplementary Table S4, available at https://doi.org/10.1016/j.esmoop.2025.105539) than patients without lung cancer. Different patient and clinical characteristics were shown to contribute to associations per cancer type (Supplementary Tables S5-S9, available at https://doi.org/10.1016/j.esmoop.2025.105539). Increasing age increased the odds of acquiring CHIP mutations and mCAs for patients with breast, rectal, and lung cancers. Not smoking or having quit smoking appeared to be protective against acquiring CHIP mutations in patients with rectal cancer, whereas it had no significant effect for patients with lung cancer. On the other hand, male sex increased the odds of harboring CHIP mutations for those with lung cancer.

### CHIP and cardiovascular endpoints

Compared with participants without CHIP mutations, those with any CHIP mutations had a higher risk of developing incident CVD [66% versus 58%, Supplementary Figure S6, available at https://doi.org/10.1016/j.esmoop.2025.105539, hazard ratio (HR) 1.07, 95% CI 1.02-1.13, *P* = 0.004, Supplementary Table S10, available at https://doi.org/10.1016/j.esmoop.2025.105539] and overall mortality (32% versus 22%, Supplementary Figure S6, available at https://doi.org/10.1016/j.esmoop.2025.105539, HR 1.31, 95% CI 1.22-1.40, *P* < 0.001, Supplementary Table S10, available at https://doi.org/10.1016/j.esmoop.2025.105539). Risks were greater for patients with expanded CHIP mutations for incident CVD (HR 1.13, 95% CI 1.07-1.20, *P* < 0.001) and for overall mortality (HR 1.42, 95% CI 1.31-1.55, *P* < 0.001, Supplementary Table S11, available at https://doi.org/10.1016/j.esmoop.2025.105539). Moreover, those with three or more CHIP mutations had higher risks of incident CVD (HR 1.24, 95% CI 1.04-1.47, *P* = 0.017), incident CAD (HR 1.36, 95% CI 1.01-1.82, *P* = 0.041), death from CVD causes (HR 2.28, 95% CI 1.22-4.26, *P* = 0.010), death from CAD causes (HR 2.40, 95% CI 0.99-5.84, *P* = 0.053), and death from any cause (HR 1.67, 95% CI 1.33-2.08, *P* < 0.001).

### CHIP co-occurring with mCAs and cardiovascular endpoints

Compared with participants without any CH, those with CHIP mutations alone (no mCAs) had a significantly higher risk of incident CVD (HR 1.08, 95% CI 1.02-1.15, *P* = 0.007). This elevated risk was no longer seen in individuals with both CHIP and mCAs (HR 1.05, 95% CI 0.96-1.15, *P* = 0.312), mirroring the null association observed in those with mCAs alone (HR 1.00, 95% CI 0.97-1.03, *P* = 0.737, Table 2). In contrast, patients with only CHIP mutations did not show an increased risk of death from CAD causes (HR 0.80, 95% CI 0.45-1.43, *P* = 0.447), whereas those with only mCAs had a higher risk compared with patients with neither CHIP nor mCA (HR 1.36, 95% CI 1.08-1.72, *P* = 0.008, Table 2).

**Table 2:** Multivariable Cox regression models assessing the risk CHIP and mCA on various cardiovascular-related endpoints

Characteristic	*N*	Event *N*	HR	95% CI	*P* value
Time to incident CVD					
No CH	36 777	20 130	Ref.	—	—
CHIP & mCA	687	484	1.048	0.957-1.148	0.312
CHIP only	1988	1270	1.082	1.022-1.145	0.007
mCA only	9410	6215	0.995	0.965-1.025	0.737
CHIP × mCA	—	—	0.974	0.874-1.086	0.635
RERI (95% CI) = –0.028 (–0.121 to 0.065)					
AP (95% CI) = –0.027 (–0.029 to –0.025)					
SI (95% CI) = 0.974 (0.890-1.058)					
Time to incident CAD					
No CH	36 777	4626	Ref.	—	—
CHIP & mCA	687	154	1.069	0.909-1.256	0.422
CHIP only	1988	338	1.110	0.994-1.240	0.065
mCA only	9410	1923	1.031	0.975-1.091	0.281
CHIP × mCA	—	—	0.933	0.766-1.138	0.494
RERI (95% CI) = –0.073 (–0.240 to 0.094)					
AP (95% CI) = –0.068 (–0.079 to –0.058)					
SI (95% CI) = 0.933 (0.792-1.074)					
Time to CV death					
No CH	36 777	507	Ref.	—	—
CHIP & mCA	687	18	1.090	0.679-1.752	0.720
CHIP only	1988	38	1.073	0.771-1.494	0.676
mCA only	9410	242	1.148	0.976-1.351	0.095
CHIP × mCA	—	—	0.885	0.495-1.584	0.681
RERI (95% CI) = –0.131 (–0.626 to 0.364)					
AP (95% CI) = –0.120 (–0.172 to –0.068)					
SI (95% CI) = 0.885 (0.500-1.270)					
Time to CAD death					
No CH	36 777	211	Ref.	—	—
CHIP & mCA	687	9	1.210	0.618-2.372	0.578
CHIP only	1988	12	0.798	0.445-1.429	0.447
mCA only	9410	132	1.363	1.083-1.716	0.008
CHIP × mCA	—	—	1.113	0.456-2.718	0.813
RERI (95% CI) = 0.050 (–0.690 to 0.790)					
AP (95% CI) = 0.041 (0.018-0.064)					
SI (95% CI) = 1.113 (0.495-1.732)					
Time to any death					
No CH	36 777	7185	Ref.	—	—
CHIP & mCA	687	268	1.406	1.243-1.591	<0.001
CHIP only	1988	591	1.305	1.200-1.420	<0.001
mCA only	9410	2596	1.068	1.018-1.121	0.007
CHIP × mCA	—	—	1.001	0.867-1.173	0.916
RERI (95% CI) = 0.032 (–0.114, 0.179)					
AP (95% CI) = 0.023 (0.021-0.025)					
SI (95% CI) = 1.008 (0.920-1.097)					

Models adjusted for age at baseline, sex, smoking status, chemotherapy, radiotherapy, prevalent CVD, number of days between date of recruitment and date of cancer diagnosis, and genotyping principal components 1-10.

AP, attributable proportion; CAD, coronary artery disease; CH, clonal hematopoiesis; CHIP, clonal hematopoiesis of indeterminate potential; CI, confidence interval; CVD, cardiovascular disease; HR, hazard ratio; mCA, mosaic chromosomal alterations; Ref., referent category; RERI, relative excess risk due to interaction; SI, synergy index.

The risk of all-cause mortality showed a dose-response relationship with the presence of CHIP and mCA. While each type of CH independently increased the risk, the combination of both both CHIP and mCA was correlated with the greatest increase in mortality risk (HR 1.41, 95% CI 1.24-1.59, *P* = 0.002). This significant association was further observed with the presence of expanded CHIP mutations alongside mCAs (Supplementary Table S12, available at https://doi.org/10.1016/j.esmoop.2025.105539), as well as CHIP mutations with expanded mCAs (Supplementary Table S13, available at https://doi.org/10.1016/j.esmoop.2025.105539). However, there was no significant interaction effect between CHIP and mCA (RERI, AP, and SI) on the risk of various cardiovascular endpoints or overall death. In our exploration of LOY or LOX (Tables 3 and 4 Figures S7-S9, available at https://doi.org/10.1016/j.esmoop.2025.105539), we found that the combination of CHIP mutations with LOX had a 2.11-fold increased risk in CAD mortality (95% CI 1.20-3.71, *P* = 0.009).

**Table 3:** Multivariable Cox regression models assessing the risk CHIP and mosaic loss of the Y chromosome in men on various cardiovascular-related endpoints

Characteristic	*N*	Event *N*	HR^1^	95% CI^1^	*P* value
Time to incident CVD					
No CH	14 812	9464	Ref.	—	—
Both	341	254	0.994	0.877-1.127	0.926
CHIP only	1032	735	1.083	1.004-1.168	0.040
LOY only	6193	4343	0.993	0.957-1.031	0.717
CHIP × LOY	—	—	0.916	0.798-1.050	0.208
RERI (95% CI) = –0.091 (–0.214, 0.031)					
AP (95% CI) = –0.094 (–0.107, –0.082)					
SI (95% CI) = 0.916 (0.798-1.034)					
Time to incident CAD					
No CH	14 812	2920	Ref.	—	—
Both	341	80	0.918	0.734-1.147	0.452
CHIP only	1032	256	1.106	0.973-1.258	0.124
LOY only	6193	1467	1.016	0.953-1.084	0.619
CHIP × LOY	—	—	0.796	0.622-1.019	0.070
RERI (95% CI) = –0.229 (–0.439 to –0.019)					
AP (95% CI) = –0.258 (–0.323 to –0.194)					
SI (95% CI) = 0.796 (0.596-0.996)					
Time to CV death					
No CH	14 812	305	—	—	
Both	341	10	0.998	0.530-1.880	0.995
CHIP only	1032	30	1.151	0.789-1.677	0.466
LOY only	6193	173	1.046	0.864-1.267	0.642
CHIP × LOY	—	—	0.360	0.445-1.823	0.771
RERI (95% CI) = –0.105 (–0.724 to 0.513)					
AP (95% CI) = –0.101 (–0.179 to –0.041)					
SI (95% CI) = 0.900 (0.307-1.494)					
Time to CAD death					
No CH	14 812	159	Ref.	—	—
Both	341	4	0.807	0.298-2.188	0.674
CHIP only	1032	12	0.902	0.500-1.625	0.730
LOY only	6193	96	1.166	0.898-1.512	0.249
CHIP × LOY	—	—	0.775	0.254-2.367	0.654
RERI (95% CI) = –0.230 (–1.094 to 0.633)					
AP (95% CI) = –0.306 (–0.709 to 0.098)					
SI (95% CI) = 0.775 (–0.249 to 1.800)					
Time to any death					
No CH	14 812	3402	Ref.	—	—
Both	341	136	1.307	1.099-1.553	0.002
CHIP only	1032	367	1.342	1.205-1.496	<0.001
LOY only	6193	1859	1.080	1.019-1.145	0.010
CHIP × LOY	—	—	0.907	0.750-1.097	0.315
RERI (95% CI) = –0.111 (–0.306 to 0.083)					
AP (95% CI) = –0.086 (–0.098 to –0.075)					
SI (95% CI) = 0.907 (0.786-1.028)					

Models adjusted for age at baseline, smoking status, chemotherapy, radiotherapy, prevalent CVD, number of days between date of recruitment and date of cancer diagnosis, and genotyping principal components 1-10.

AP, attributable proportion; CAD, coronary artery disease; CH, clonal hematopoiesis; CHIP, clonal hematopoiesis of indeterminate potential; CI, confidence interval; CVD, cardiovascular disease; HR, hazard ratio; LOY, mosaic loss of the Y chromosome; mCA, mosaic chromosomal alteration; Ref., referent category; RERI, relative excess risk due to interaction; SI, synergy index.

**Table 4:** Multivariable Cox regression models assessing the risk CHIP and mosaic loss of the X chromosome in women on various cardiovascular-related endpoints

Characteristic	*N*	Event *N*	HR	95% CI	*P* value
Time to incident CVD					
No CH	23 649	11 678	Ref.	—	—
Both	119	73	1.169	0.928-1.472	0.185
CHIP only	1183	692	1.090	1.009-1.177	0.028
LOX only	1533	860	1.011	0.943-1.084	0.749
CHIP × LOX	—	—	1.072	0.840-1.369	0.574
RERI (95% CI) = 0.082 (–0.168 to 0.333)					
AP (95% CI) = 0.069 (0.056-0.083)					
SI (95% CI) = 1.072 (0.864-1.281)					
Time to incident CAD					
No CH	23 649	1970	—	—	
Both	119	18	1.298	0.815-2.068	0.272
CHIP only	1183	138	1.209	1.017-1.438	0.031
LOX only	1533	192	1.201	1.034-1.394	0.016
CHIP × LOX	—	—	0.963	0.589-1.576	0.881
RERI (95% CI) = –0.029 (–0.558 to 0.500)					
AP (95% CI) = –0.022 (–0.031 to –0.014)					
SI (95% CI) = 0.963 (0.618-1.308)					
Time to CV death					
No CH	23 649	244	—	—	
Both	119	0	0.000	0.000-Inf	0.988
CHIP only	1183	16	1.053	0.634-1.748	0.843
LOX only	1533	27	1.248	0.836-1.863	0.278
CHIP × LOX	—	—	nr	—	0.978
RERI (95% CI) = –1.348 (–2.749 to 0.032)					
AP (95% CI) = nr					
SI (95% CI) = nr					
Time to CAD death					
No CH	23 649	73	Ref.	—	—
Both	119	0	0.000	0.000-Inf	0.994
CHIP only	1183	5	1.086	0.438-2.692	0.859
LOX only	1533	15	2.111	1.203-3.705	0.009
CHIP × LOX	—	—	nr	—	0.986
RERI (95% CI) = nr					
AP (95% CI) = nr					
SI (95% CI) = nr					
Time to any death					
No CH	23 649	4209	Ref.	—	—
Both	119	40	1.470	1.076-2.009	0.016
CHIP only	1183	316	1.310	1.167-1.469	<0.001
LOX only	1533	311	0.985	0.877-1.106	0.801
CHIP × LOX	—	—	1.13	0.807-1.583	0.477
RERI (95% CI) = 0.168 (–0.209 to 0.546)					
AP (95% CI) = 0.115 (0.090-0.139)					
SI (95% CI) = 1.130 (0.890-1.370)					

Models adjusted for age at baseline, smoking status, chemotherapy, radiotherapy, prevalent CVD, number of days between date of recruitment and date of cancer diagnosis, and genotyping principal components 1-10.

AP, attributable proportion; CAD, coronary artery disease; CH, clonal hematopoiesis; CHIP, clonal hematopoiesis of indeterminate potential; CI, confidence interval; CVD, cardiovascular disease; HR, hazard ratio; Inf, infinity, LOX, mosaic loss of the X chromosome; mCA, mosaic chromosomal alteration; nr, not reportable due to small number of events; Ref., referent category; RERI, relative excess risk due to interaction; SI, synergy index.

### CHIP and cardiovascular endpoints per cancer type

*P* values derived from CHIP-by-cancer type interaction terms Cox regression analyses specific to cancer types showed that some of our associations between CHIP and outcomes differed by tumor type (Supplementary Tables 14 to 22, available at https://doi.org/10.1016/j.esmoop.2025.105539). For example, women with corpus uteric cancer with CHIP mutations had a higher risk of incident CVD (HR 1.41, 95% CI 1.13-1.77, *P*_interaction_ = 0.024) and CAD (HR 2.31, 95% CI 1.50-3.56, *P*_interaction_ = 0.001, Supplementary Table 16, available at https://doi.org/10.1016/j.esmoop.2025.105539) than those without CHIP mutations.

## Discussion

In this study, we examined whether carriage of CHIP mutations was associated with cardiovascular-related endpoints in cancer patients within the UK Biobank. Specifically, patients with CHIP mutations face significantly increased risks of both incident CVD and all-cause mortality, especially those carrying larger clones. CHIP and mCAs independently influenced distinct clinical endpoints: isolated CHIP showed a stronger association with new CVD events, whereas isolated mCAs more closely predicted CAD-related death. Although carriers of both CHIP and mCAs experienced the highest overall mortality, there was no evidence of a synergistic interaction between these two forms of clonal hematopoiesis on any of the outcomes evaluated.

CH, an age-related expansion of mutated blood cells, is an established marker for immune dysregulation, increased inflammatory disease, and hematologic cancer risk.^24^, ^25^, ^26^, ^27^ A landmark study of 17 182 individuals without prevalent hematologic cancers found that somatic CHIP mutations increase the risk of developing hematologic malignancies by 11-fold and to increase all-cause mortality by 1.4-fold, largely driven by cardiovascular causes.^5^ The unexpected finding that CH also contributes to CVD suggests that CH can play a crucial role in predicting short- and long-term cardiovascular events. Patients diagnosed with cancer have a notably higher risk of dying from atherosclerosis,^9^^,^^10^ potentially due to therapy-induced cardiovascular complications^12^^,^^28^^,^^29^ perpetuated by overlapping biological processes of both disease phenotypes.^30^ Despite recent breakthroughs in cancer treatments reducing cancer-related deaths, CVD remains the most frequent cause of death among long-term survivors of various cancers.^31^^,^^32^ Therefore, identifying cancer patients or survivors who might benefit from more intensive cardiovascular monitoring would be ideal. However, conventional cardiac biomarkers used in baseline cardiovascular assessments appear inadequate.^33^ Given that CH intersects cancer and cardiovascular risk while implicating inflammation, there is a significant opportunity to leverage CH as a tool for precision oncology. Hence, in this study, we sought to evaluate the impact of CH on short- and long-term cardiovascular outcomes in a large cohort of patients diagnosed with cancer.

In a previous study, individuals with both CHIP and mCAs were shown to have increased clonal expansion^34^ and risk of leukemic transformation indicating a synergistic impact on carcinogenesis.^35^ Here, we found that individuals with small cell fraction mCAs were not at higher odds for the presence of CHIP mutations. However, those with larger cell fraction (>10%) mCAs had higher odds of being CHIP carriers. We observed that CHIP was inversely correlated with LOY, possibly linked to variants at the *SETBP1* locus, which are negatively associated with CHIP but positively associated with LOY.^19^ Conversely, we found that women carrying LOX were more likely to harbor CHIP mutations, indicating distinct biological processes for sex chromosome missegretation.^36^ Cancer-specific associations revealed distinct CHIP and mCA phenotypes across cancer types.

In our primary findings, we found that individuals with CHIP had a higher risk of incident CVD (HR 1.07), with increased effect sizes for CHIP mutations with larger VAF (≥0.10) (HR 1.13), consistent with a previous study (expanded CHIP on CVD risk: HR 1.11, *n* = 628 388).^19^ However, we found a lower HR for CHIP carriage and incident CVD compared with a previous report^37^ (HR 1.59). This discrepancy may be due to that study’s estimate being based on only the first 50 000 participants of the UK Biobank and on their CVD phenotypic definition, which included all-cause mortality.^37^ When examining the effect of CHIP on all-cause mortality, we reported a risk estimate of 1.31 for all CHIP mutations, increasing to 1.42 for those with expanded CHIP mutations. This difference in mortality was also observed for all mCAs and expanded mCAs. When CH expands to involve a large fraction of blood cells, the clonality of abnormal cells drive chronic inflammation and worsen immune defenses, which then increase cardiovascular events and hematologic cancer, ultimately raising overall mortality.^7^^,^^17^^,^^38^

The combined effect of CHIP mutations and mCA was detrimental only when considering overall mortality, where having both forms of CH led to the highest risk of all-cause mortality (HR_CHIP+mCA_ 1.41). Interaction term analyses suggested that CHIP and mCA presence did not synergistically impact risk beyond individual effects. Some (RERI, AP, SI) even indicated slight antagonistic effects, but not sufficiently statistically robust to draw definitive conclusions.

In subanalyses examining the impact of CH on cardiovascular outcomes for specific cancers, we found that the presence of CHIP mutations was linked to adverse cardiovascular events and increased mortality in women diagnosed with breast cancer and corpus uteri cancer. Previously understudied, it is now evident that certain forms of CH, along with LOX, serve as important preclinical indicators of hematologic malignancies, autoimmune dysfunction, and cancer predisposition in women.^36^ Consequently, our findings, along with previous data, reiterate the need for ongoing research into the clinical significance of CH in women’s health.

Our study has several limitations. Firstly, its retrospective design precludes causal inference. Secondly, we lacked granular tumor and treatment data—stage, metastatic status, therapy type, and duration—which hampers interpretation. CH prevalence, likely owing to age-related clonal dynamics, varied by cancer type. Not surprisingly, the association between CHIP and CV outcomes differed according to cancer type. This was evidenced by significant CHIP-by-cancer type interaction tests in the overall population. Subgroup estimates in patients diagnosed with specific cancers unfortunately lacked statistical power to more accurately capture true effects. At the same time, we could not determine whether these differences stem from intrinsic biology or therapy-induced CH. Finally the absence of stage information prevents precise adjustment for disease burden, so the effect of CH on overall survival may be under- or overestimated.^39^^,^^40^

The one-time abstraction of CH status at study entry prevented the evaluation of clonal expansion over time. The UK Biobank’s younger cohort may have had unmeasurable CH at study entry, which expanded during follow-up. The patient cohort represents an earlier generation of cancer patients, limiting analysis of contemporary treatment impacts, including tyrosine kinase inhibitors and immune checkpoint blockade drugs.^41^ Previously it has been shown that CH mutations in the DNA damage repair (DDR) genes (i.e. *TP53*, *PPM1D*, and *CHEK2*) which are selected under exposure to chemotherapy are more prevalent than mutations in genes involved in epigenetic modifiers (i.e. *DMNT3A*, *TET2*) or splicing regulators (i.e. *SRSF2*, *U2AF1*).^41^ Our results could not reliably confirm this as time of chemotherapy was unavailable. However, *PPM1D* and *TP53* were indeed more frequent in those exposed to chemotherapy. Subanalyses on combinations of specific CHIP mutations and mCA classes (LOX, LOY) on clinical outcomes were exploratory and lacked sufficient power for robust interpretation, but suggested distinct outcomes for different CH combinations. Furthermore, mosaic events were limited to commonly defined groups, namely LOX, LOY, and autosomal mCAs. This gross categorization of mCAs fails to fully apprehend the various associations that other mosaic subclasses may hold with regards to health outcomes.^42^^,^^43^

In summary, cancer patients with CHIP mutations face higher risks of CVD and all-cause mortality, intensified by expanded CHIP mutations. The combination of CHIP and mCAs additively worsens overall mortality risk, underscoring the importance of considering CH in cardiovascular risk assessments for cancer patients.

## Funding

MS was supported by a doctorate scholarship from the Fonds de Recherche du Québec—Santé (FRQS). M-PD and J-CT hold Canada Research Chairs. This project was supported in part by the Health Collaboration Acceleration Fund (FACS) (no grant number) from the Government of Quebec (J-CT principal investigator and M-PD coprincipal investigator).

## Disclosure

**M-PD** reports a minor equity interest in DalCor Pharmaceuticals. **M-PD** has a patent ‘Methods for Treating or Preventing Cardiovascular Disorders and Lowering Risk of Cardiovascular Events’, issued to DalCor Pharmaceuticals, no royalties received; a patent ‘Genetic Markers for Predicting Responsiveness to Therapy with HDL-Raising or HDL Mimicking Agent’, issued to DalCor Pharmaceuticals, no royalties received; and a patent ‘Methods for Using Low Dose Colchicine After Myocardial Infarction’, assigned to the Montreal Heart Institute.

**J-CT** reports research grants from Amarin, AstraZeneca, Ceapro, DalCor Pharmaceuticals, Esperion, Ionis, Merck, Novartis, and Pfizer; honoraria from DalCor Pharmaceuticals, HLS Therapeutics, Pendopharm, and Pfizer; minor equity interest from DalCor Pharmaceuticals; authorship on a patent ‘Methods for Treating or Preventing Cardiovascular Disorders and Lowering Risk of Cardiovascular Events’, issued to DalCor Pharmaceuticals, no royalties received; a patent ‘Genetic Markers for Predicting Responsiveness to Therapy with HDL-Raising or HDL Mimicking Agent’, issued to DalCor Pharmaceuticals, no royalties received; a pending patent ‘Early Administration Of Low-Dose Colchicine After Myocardial Infarction’, and a patent ‘Methods for Using Low-Dose Colchicine After Myocardial Infarction’, assigned to the Montreal Heart Institute (**J-CT** has waived his rights in the colchicine patents and does not stand to gain financially).

All other authors have declared no conflicts of interest.
