# Supplementary material for: Impact of clonal hematopoiesis on cardiovascular outcomes in cancer patients of the UK Biobank
Source: ESMO Open. 2025 Aug 7;10(8):105539. doi: 10.1016/j.esmoop.2025.105539 (PMC12355096; doi:10.1016/j.esmoop.2025.105539)
Supplement: Supplemental Figure S1 [file mmc1.pdf]

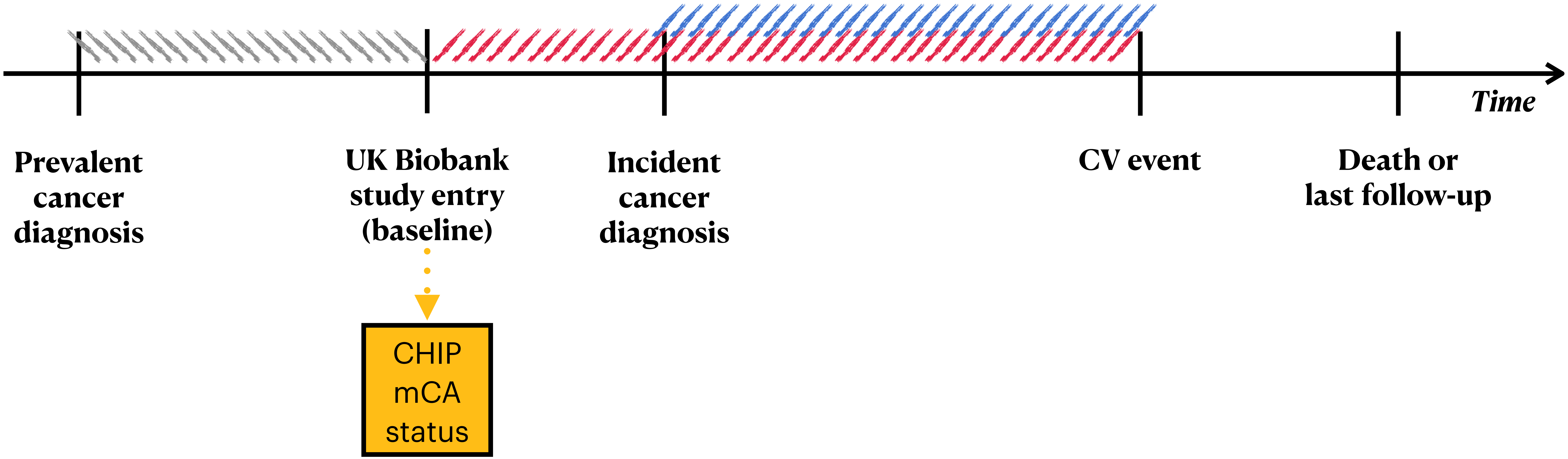

Figure 1. Illustration of time to event definitions of the current study. For patients with prevalent cancers (i.e. diagnosed before the UK Biobank study entry date), time to event represented the interval between the study entry date (baseline) and the CV event of interest **(red hashed lines)**. For patients with incident cancers (i.e. diagnosed after the UK Biobank study entry date), time to event represented the interval between the incident cancer diagnosis date and the CV event of interest **(blue hashed lines)**. For individuals who did not experience any CV event of interest, the end of follow-up was the last date of death or last date of follow-up based on the participant's county of enrolment. To overcome for 'time bias' incurred due to prevalent cancer diagnoses (left-truncating), all our models accounted for the time interval (in days) between a patient's prevalent cancer diagnosis date and the study entry date **(grey hashed lines)**, setting it to zero if the cancer diagnosis occurred after the study entry date.
