# Supplementary figures and images for: Impact of clonal hematopoiesis on cardiovascular outcomes in cancer patients of the UK Biobank
Source: ESMO Open. 2025 Aug 7;10(8):105539. doi: 10.1016/j.esmoop.2025.105539 (PMC12355096; doi:10.1016/j.esmoop.2025.105539)

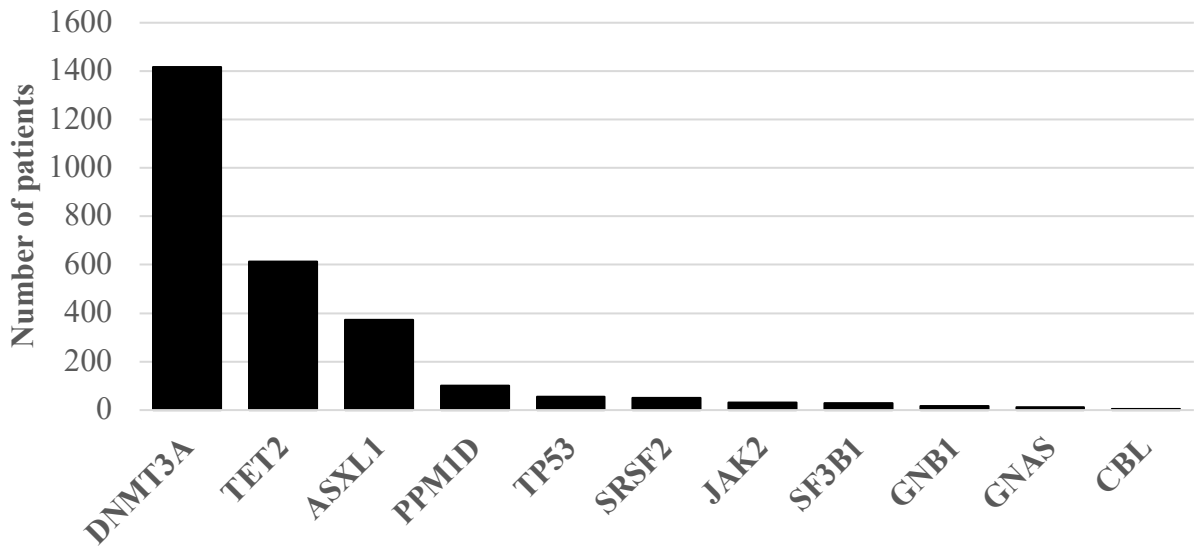

Supplement: Supplemental Figure S2 [file mmc2.pdf]

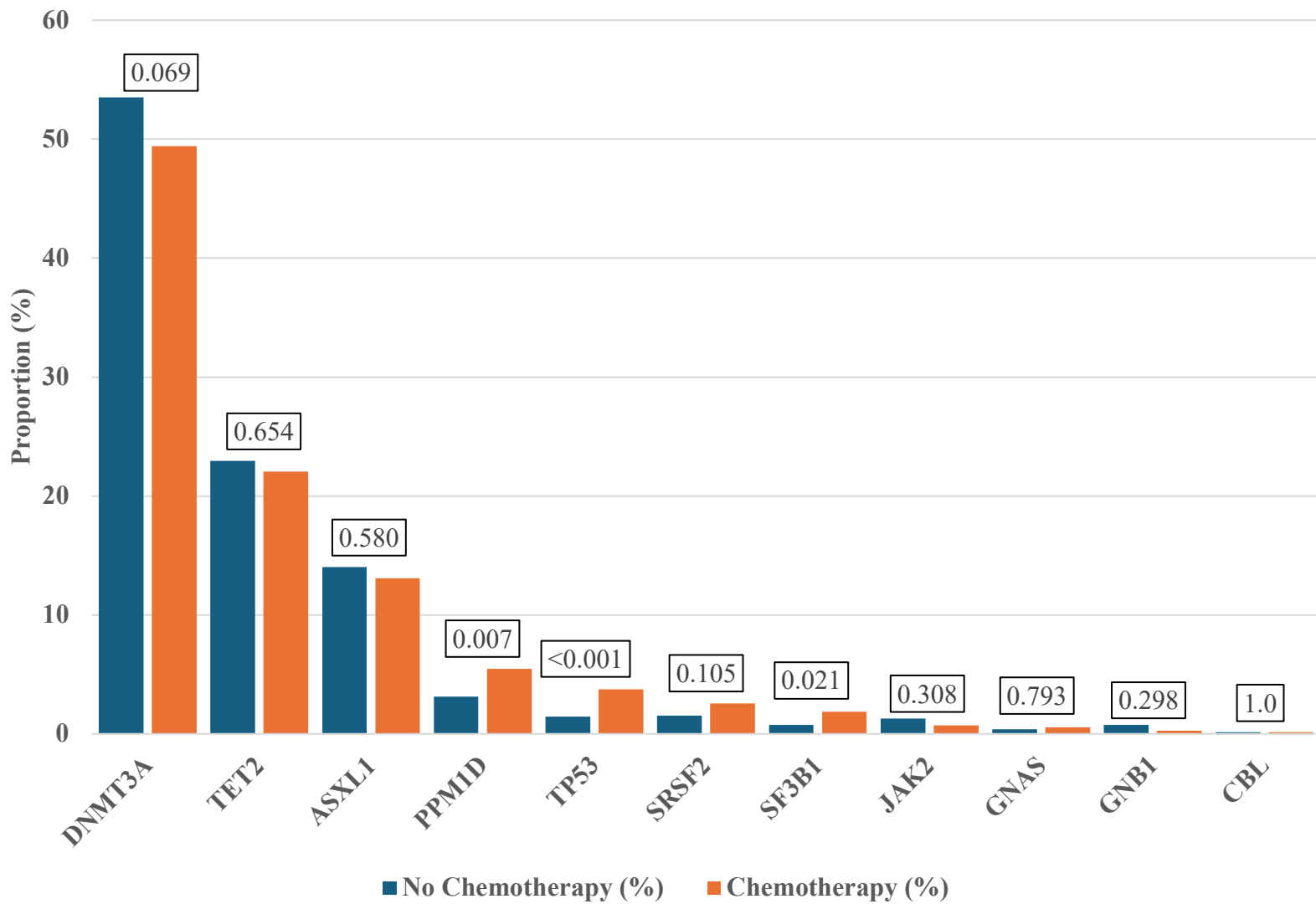

Supplement: Supplemental Figure S3 [file mmc3.pdf]

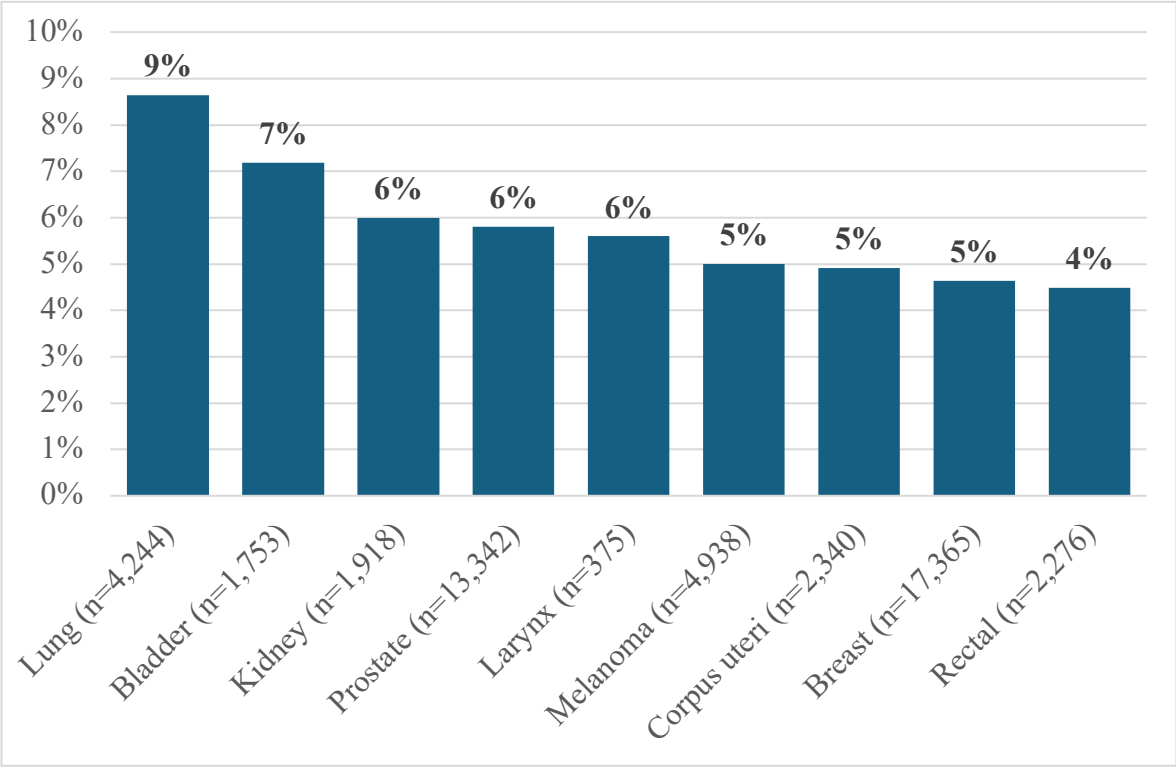

Supplement: Supplemental Figure S4 [file mmc4.pdf]

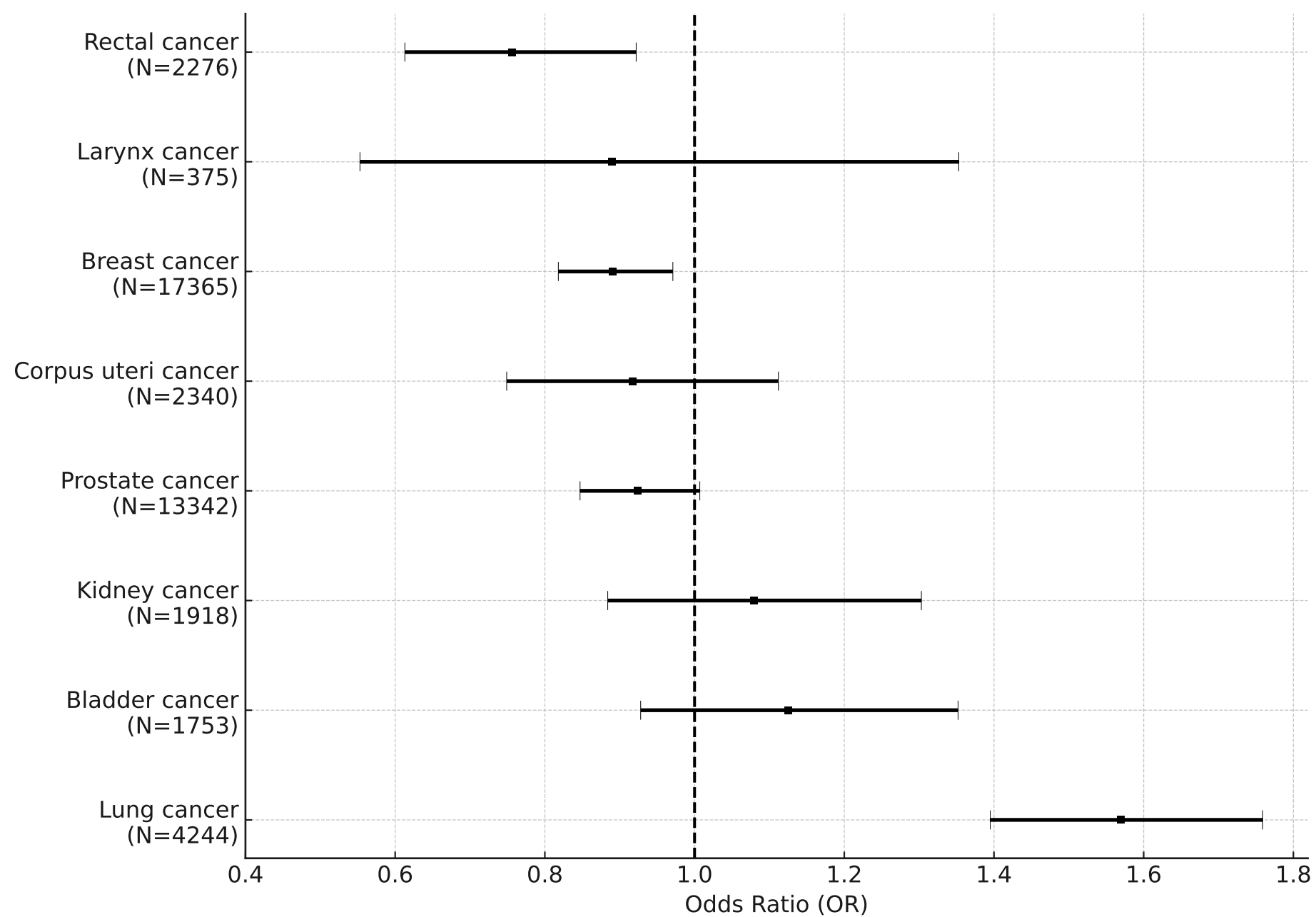

Supplement: Supplemental Figure S5 [file mmc5.pdf]

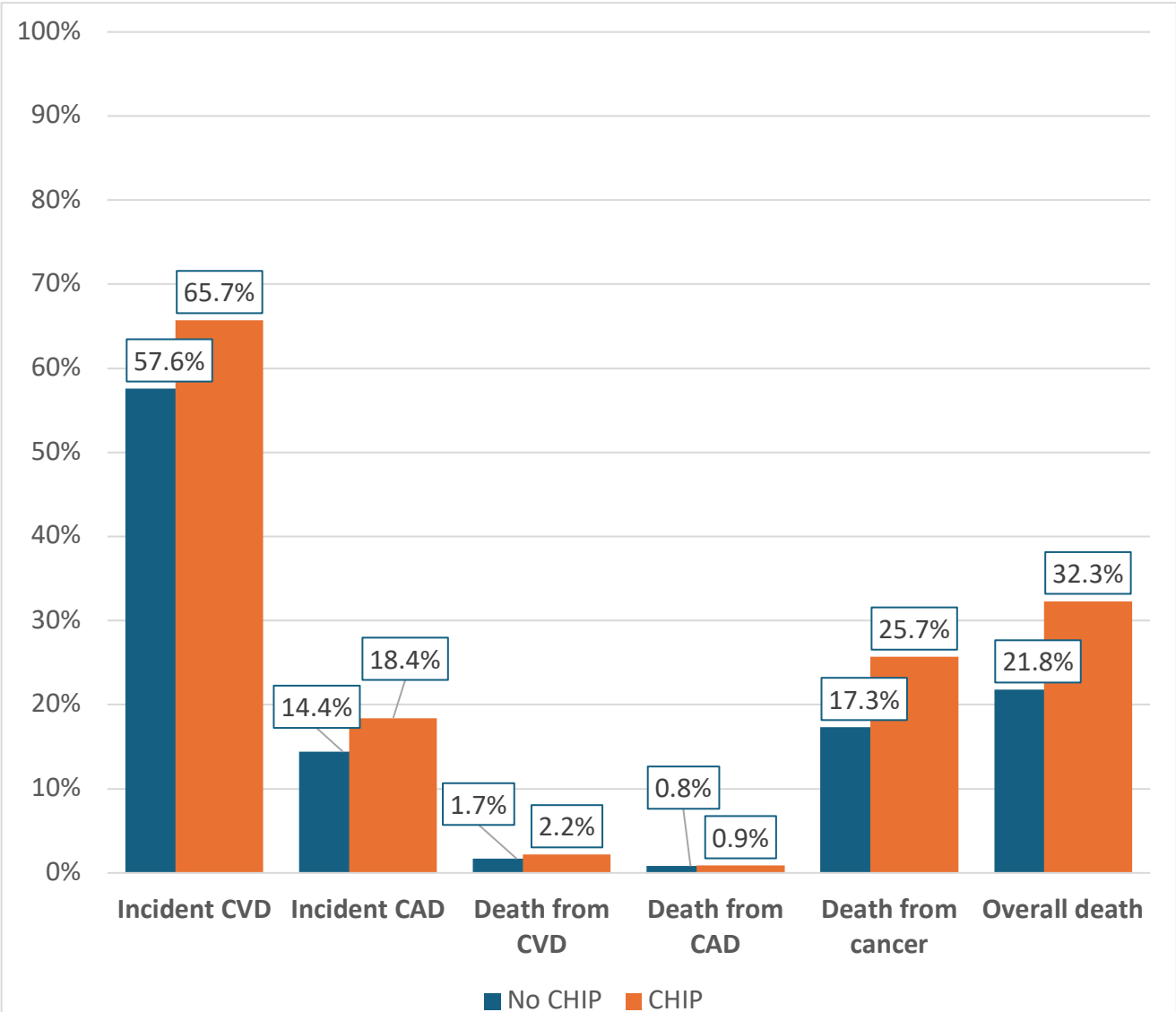

Supplement: Supplemental Figure S6 [file mmc6.pdf]
