# Supplementary material for: Impact of clonal hematopoiesis on cardiovascular outcomes in cancer patients of the UK Biobank
Source: ESMO Open. 2025 Aug 7;10(8):105539. doi: 10.1016/j.esmoop.2025.105539 (PMC12355096; doi:10.1016/j.esmoop.2025.105539)
Supplement: Supplemental Figure S7 [file mmc7.pdf]

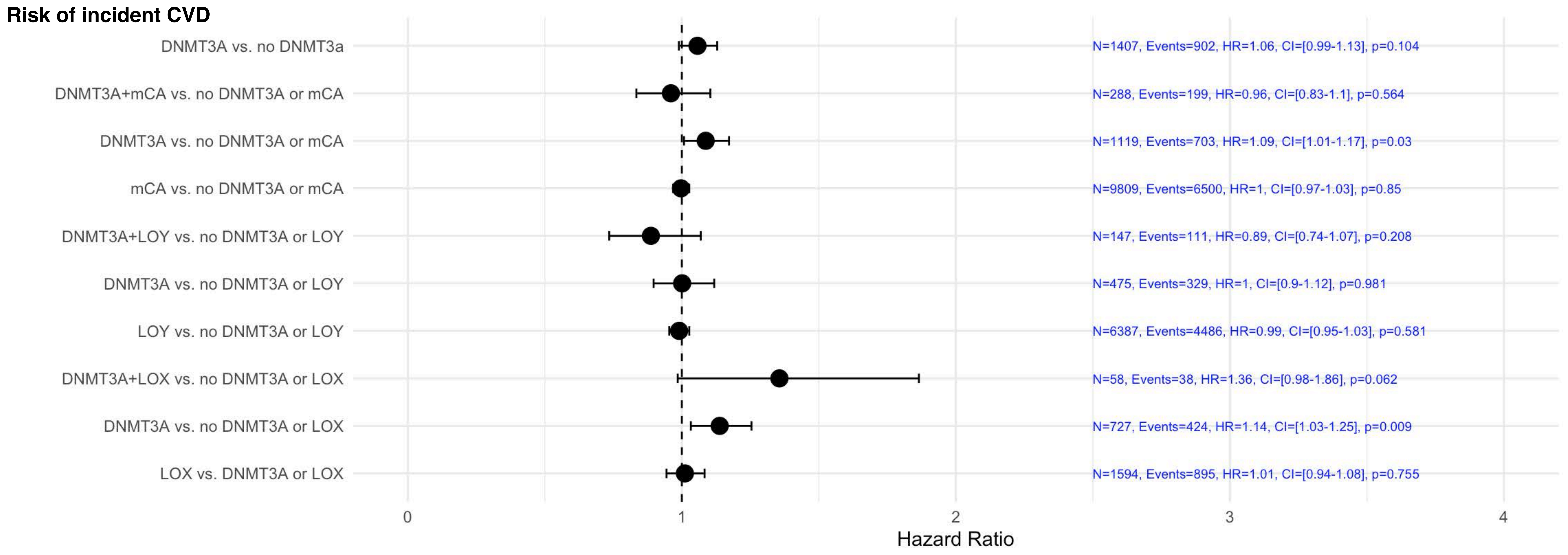
Risk of incident CVD
DNMT3A vs. no DNMT3a
DNMT3A+mCA vs. no DNMT3A or mCA
DNMT3A vs. no DNMT3A or mCA
mCA vs. no DNMT3A or mCA
DNMT3A+LOY vs. no DNMT3A or LOY
DNMT3A vs. no DNMT3A or LOY
LOY vs. no DNMT3A or LOY
DNMT3A+LOX vs. no DNMT3A or LOX
DNMT3A vs. no DNMT3A or LOX
LOX vs. DNMT3A or LOX
N=1407, Events=902, HR=1.06, CI=[0.99-1.13], p=0.104
N=288, Events=199, HR=0.96, CI=[0.83-1.1], p=0.564
N=1119, Events=703, HR=1.09, CI=[1.01-1.17], p=0.03
N=9809, Events=6500, HR=1, CI=[0.97-1.03], p=0.85
N=147, Events=111, HR=0.89, CI=[0.74-1.07], p=0.208
N=475, Events=329, HR=1, CI=[0.9-1.12], p=0.981
N=6387, Events=4486, HR=0.99, CI=[0.95-1.03], p=0.581
N=58, Events=38, HR=1.36, CI=[0.98-1.86], p=0.062
N=727, Events=424, HR=1.14, CI=[1.03-1.25], p=0.009
N=1594, Events=895, HR=1.01, CI=[0.94-1.08], p=0.755
0
1
2
3
4
Hazard Ratio

Risk of incident CAD

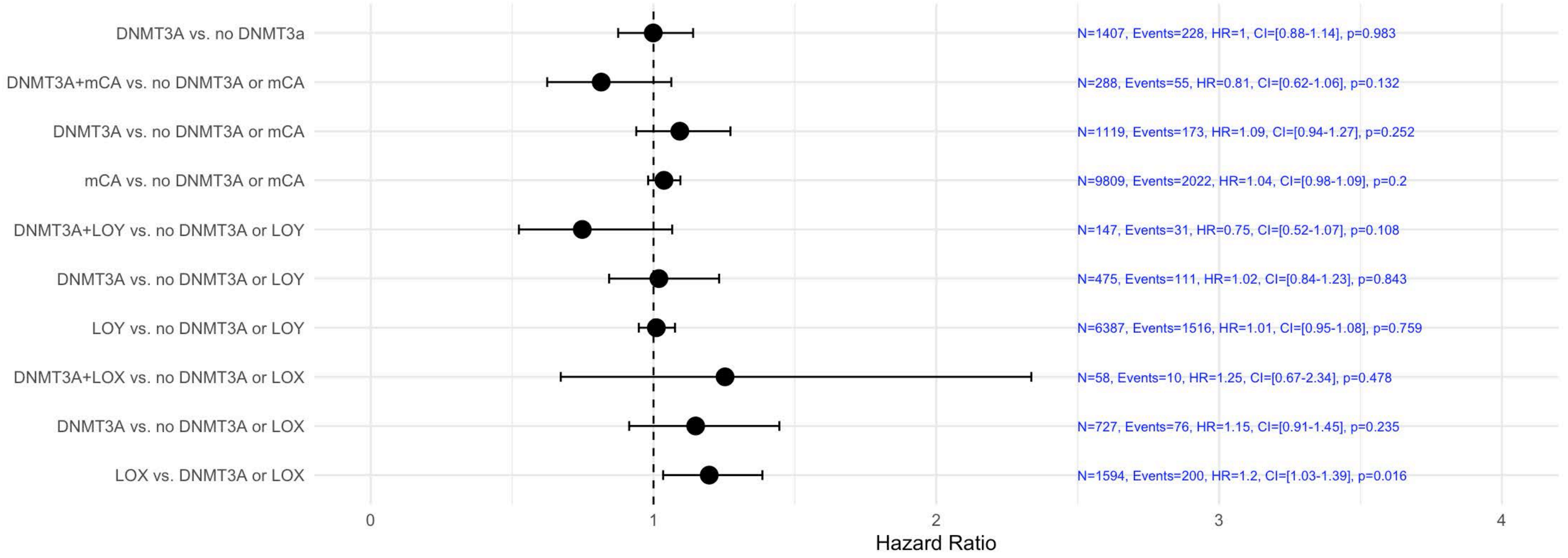

Risk of death of CVD causes

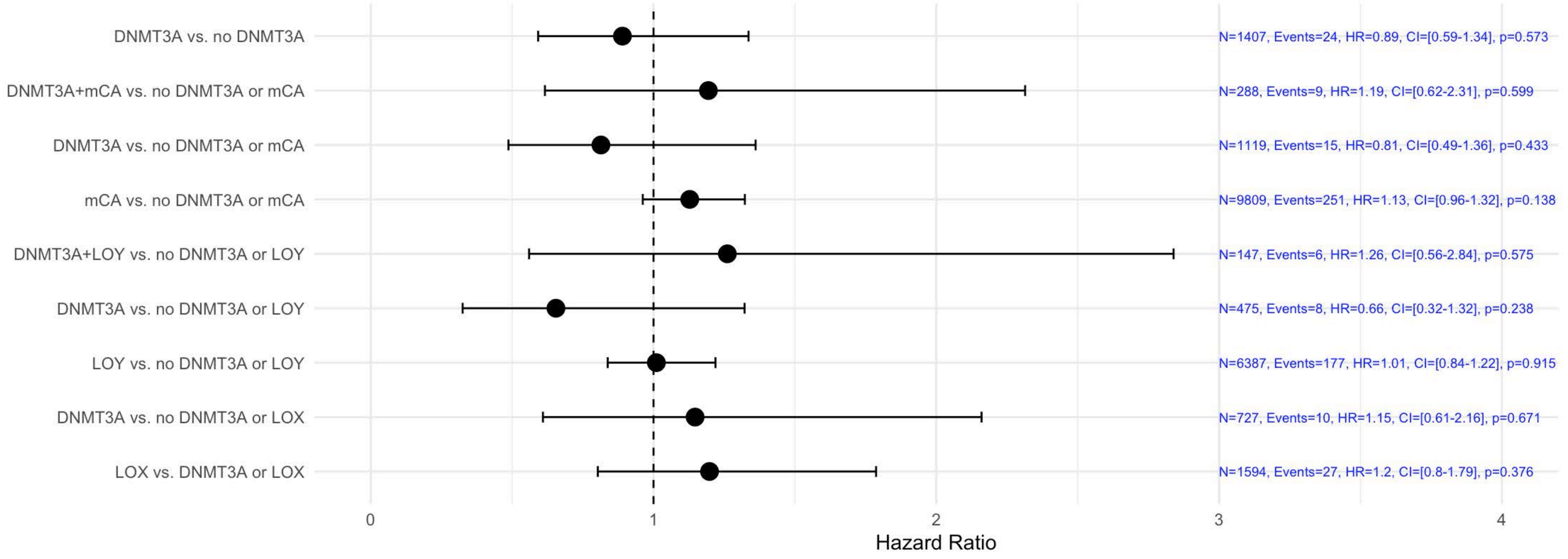

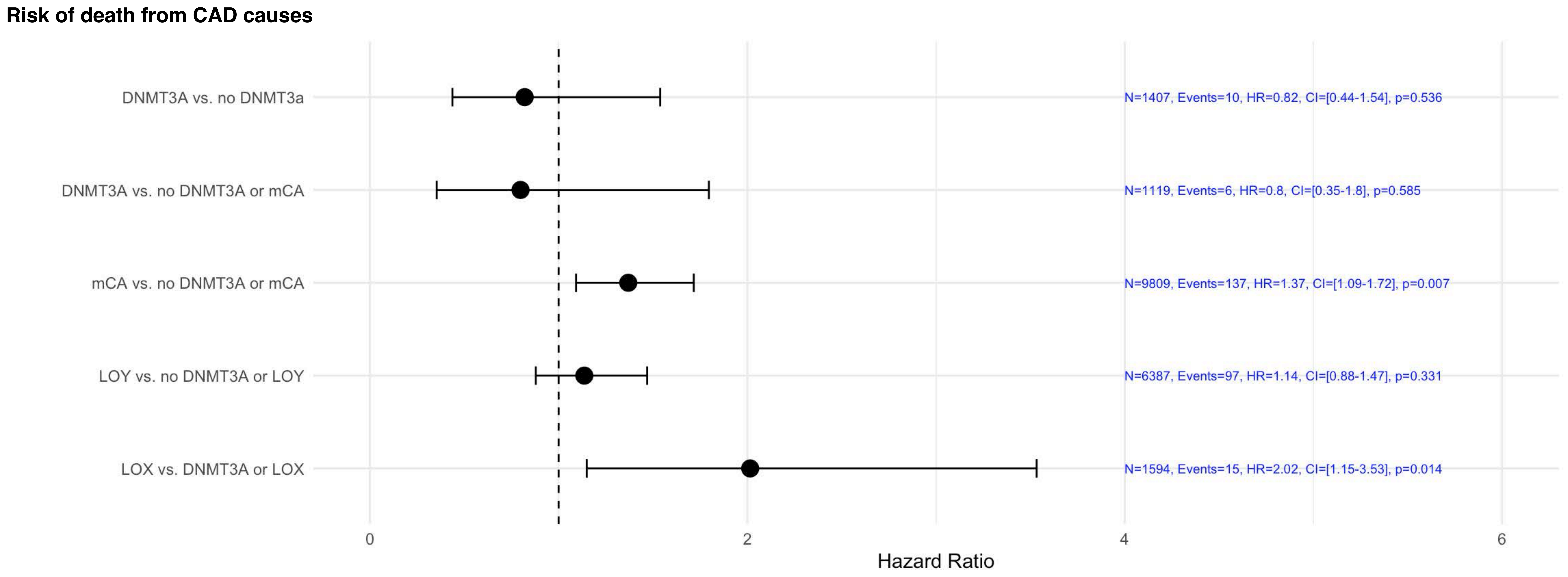
Risk of death from CAD causes
DNMT3A vs. no DNMT3a
DNMT3A vs. no DNMT3A or mCA
mCA vs. no DNMT3A or mCA
LOY vs. no DNMT3A or LOY
LOX vs. DNMT3A or LOX
N=1407, Events=10, HR=0.82, CI=[0.44-1.54], p=0.536
N=1119, Events=6, HR=0.8, CI=[0.35-1.8], p=0.585
N=9809, Events=137, HR=1.37, CI=[1.09-1.72], p=0.007
N=6387, Events=97, HR=1.14, CI=[0.88-1.47], p=0.331
N=1594, Events=15, HR=2.02, CI=[1.15-3.53], p=0.014
0
2
4
6
Hazard Ratio

Risk of overall death

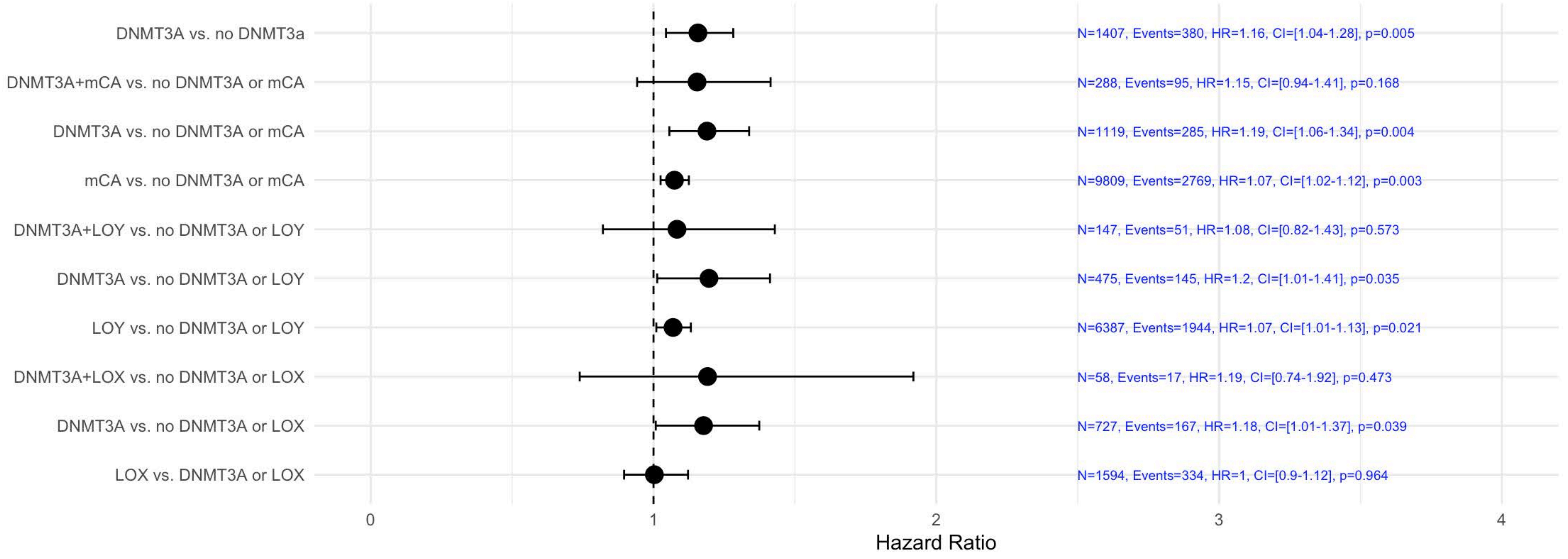

Suppl. Figure 2. Cox regression models assessing the effect of dnmt3a co-occurring with mCAs, LOY, or LOX for the risk of incident CVD [1], incident CAD [2], death of CVD causes [3], death of CAD causes [4], and any death [5]. CAD: coronary artery disease, CVD: cardiovascular disease, LOX: mosaic loss of the X chromosome, LOY: mosaic loss of the Y chromosome, mCA: mosaic chromosomal alterations
