# Supplementary material for: Impact of clonal hematopoiesis on cardiovascular outcomes in cancer patients of the UK Biobank
Source: ESMO Open. 2025 Aug 7;10(8):105539. doi: 10.1016/j.esmoop.2025.105539 (PMC12355096; doi:10.1016/j.esmoop.2025.105539)
Supplement: Supplemental Figure S8 [file mmc8.pdf]

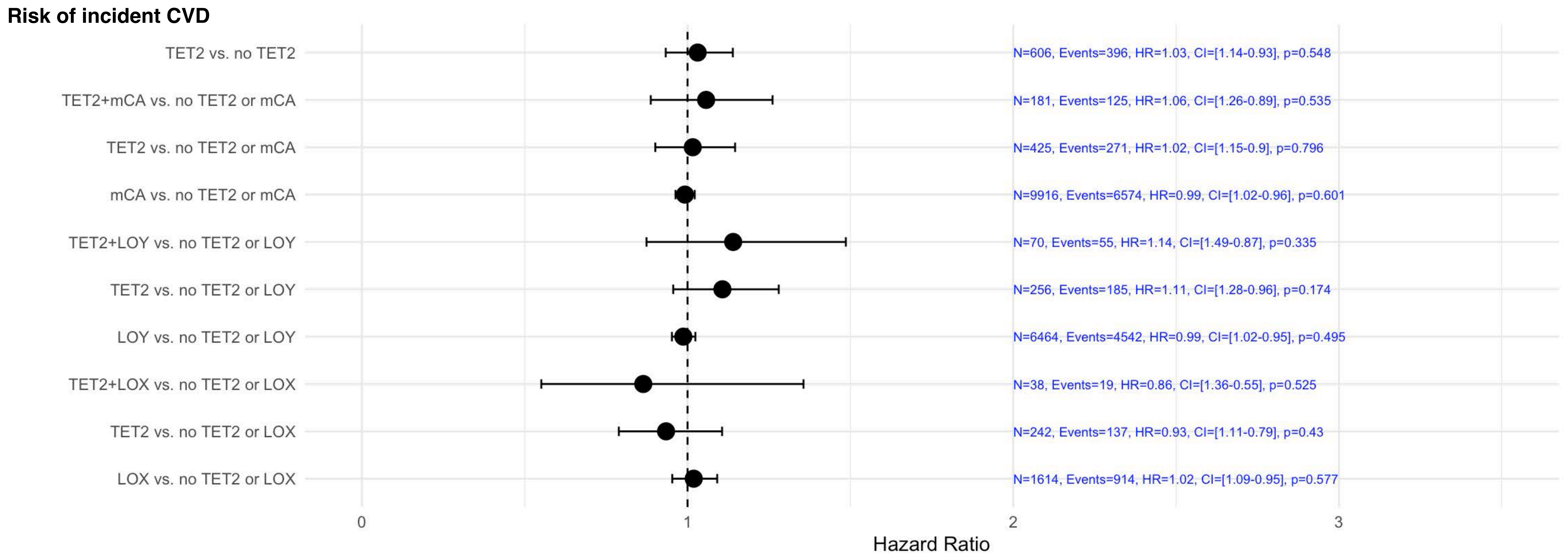
Risk of incident CVD
TET2 vs. no TET2
TET2+mCA vs. no TET2 or mCA
TET2 vs. no TET2 or mCA
mCA vs. no TET2 or mCA
TET2+LOY vs. no TET2 or LOY
TET2 vs. no TET2 or LOY
LOY vs. no TET2 or LOY
TET2+LOX vs. no TET2 or LOX
TET2 vs. no TET2 or LOX
LOX vs. no TET2 or LOX
N=606, Events=396, HR=1.03, CI=[1.14-0.93], p=0.548
N=181, Events=125, HR=1.06, CI=[1.26-0.89], p=0.535
N=425, Events=271, HR=1.02, CI=[1.15-0.9], p=0.796
N=9916, Events=6574, HR=0.99, CI=[1.02-0.96], p=0.601
N=70, Events=55, HR=1.14, CI=[1.49-0.87], p=0.335
N=256, Events=185, HR=1.11, CI=[1.28-0.96], p=0.174
N=6464, Events=4542, HR=0.99, CI=[1.02-0.95], p=0.495
N=38, Events=19, HR=0.86, CI=[1.36-0.55], p=0.525
N=242, Events=137, HR=0.93, CI=[1.11-0.79], p=0.43
N=1614, Events=914, HR=1.02, CI=[1.09-0.95], p=0.577
0
1
2
3
Hazard Ratio

Risk of incident CAD

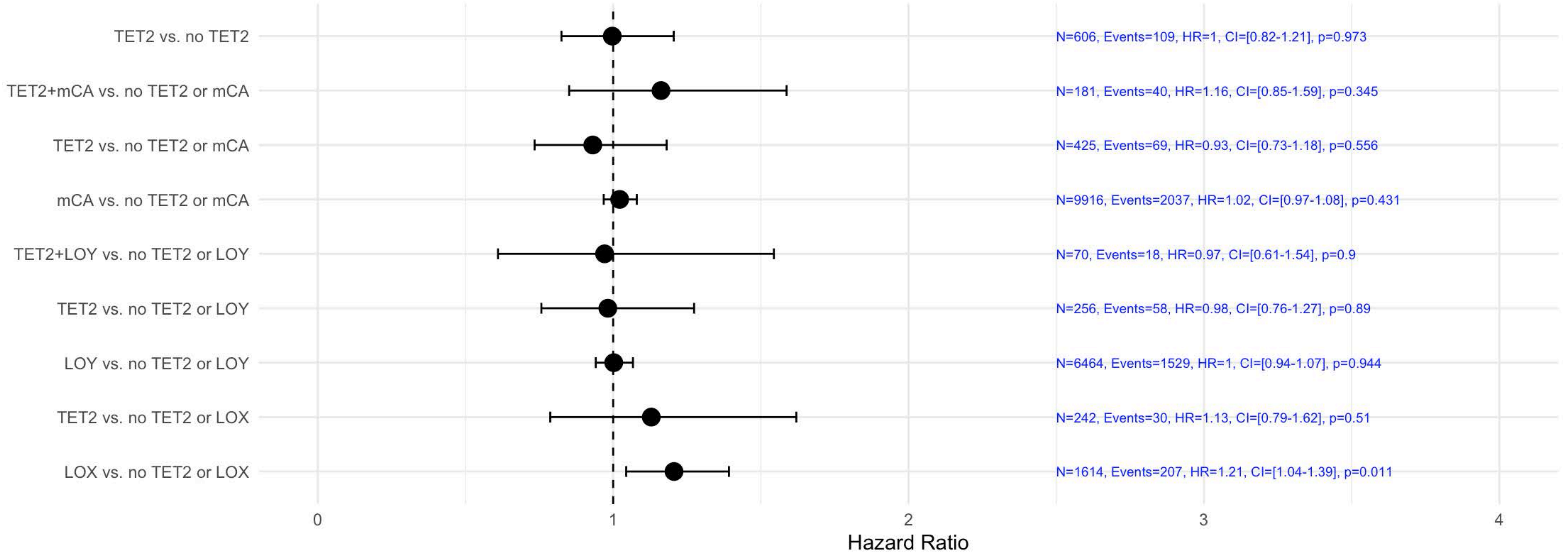

Risk of death from CVD causes

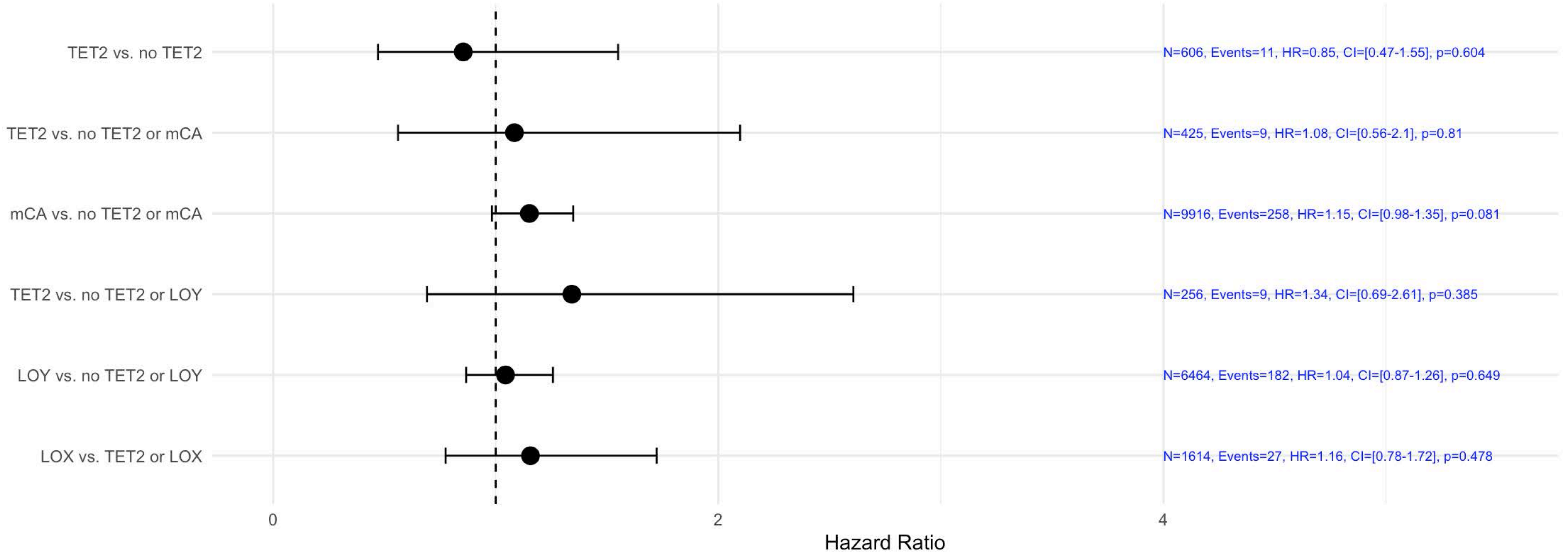

Risk of death from CAD causes

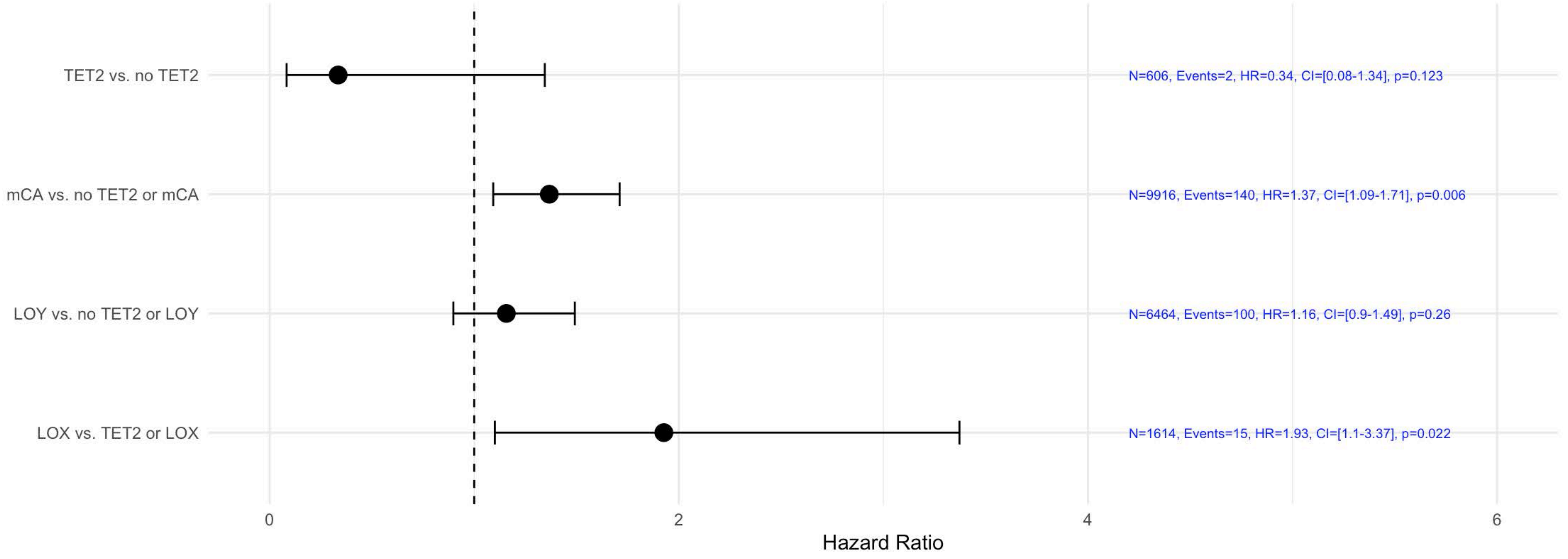

Risk of overall death

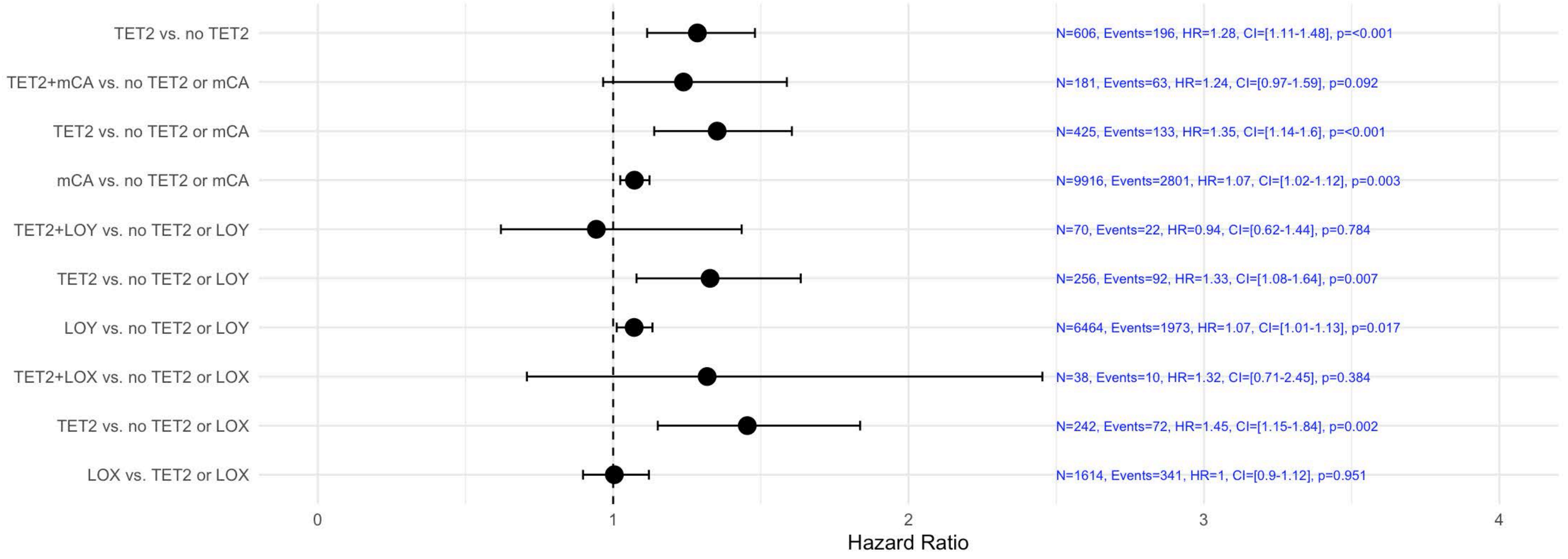

Suppl. Figure 3. Cox regression models assessing the effect of tet2 co-occurring with mCAs, LOY, or LOX for the risk of incident CVD [1], incident CAD [2], death of CVD causes [3], death of CAD causes [4], and any death [5]. CAD: coronary artery disease, CVD: cardiovascular disease, LOX: mosaic loss of the X chromosome, LOY: mosaic loss of the Y chromosome, mCA: mosaic chromosomal alterations
