# Supplementary material for: Impact of clonal hematopoiesis on cardiovascular outcomes in cancer patients of the UK Biobank
Source: ESMO Open. 2025 Aug 7;10(8):105539. doi: 10.1016/j.esmoop.2025.105539 (PMC12355096; doi:10.1016/j.esmoop.2025.105539)
Supplement: Supplemental Figure S9 [file mmc9.pdf]

Risk of incident CVD

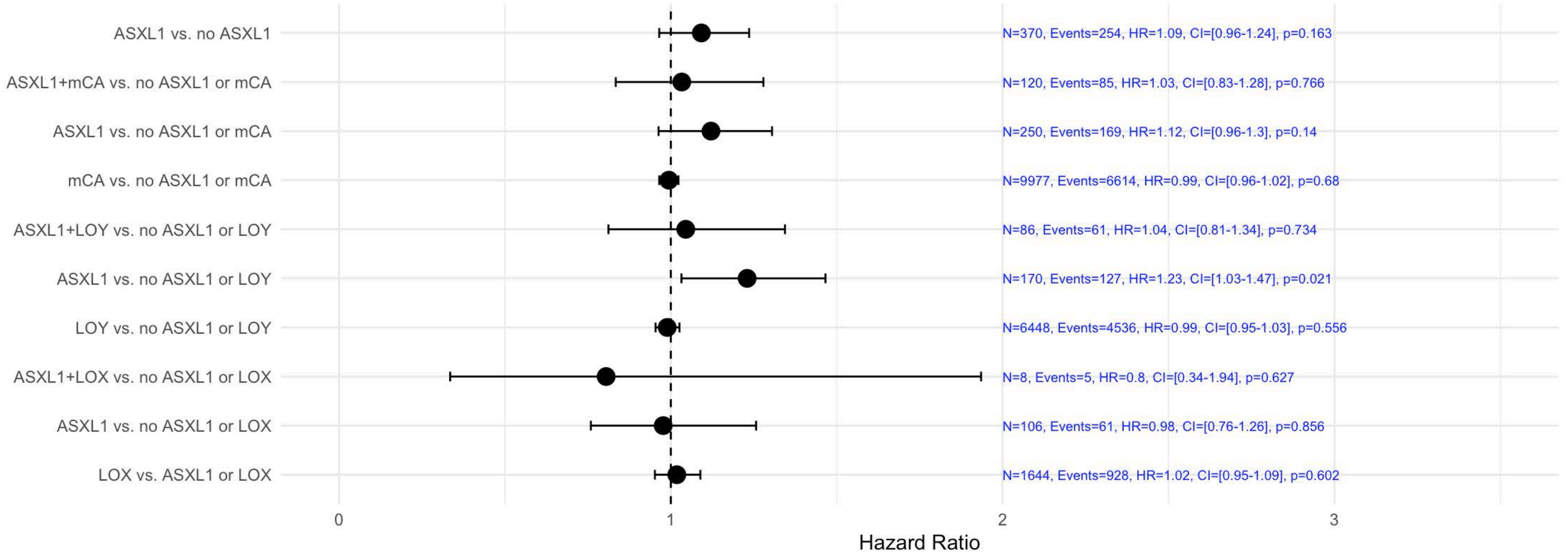

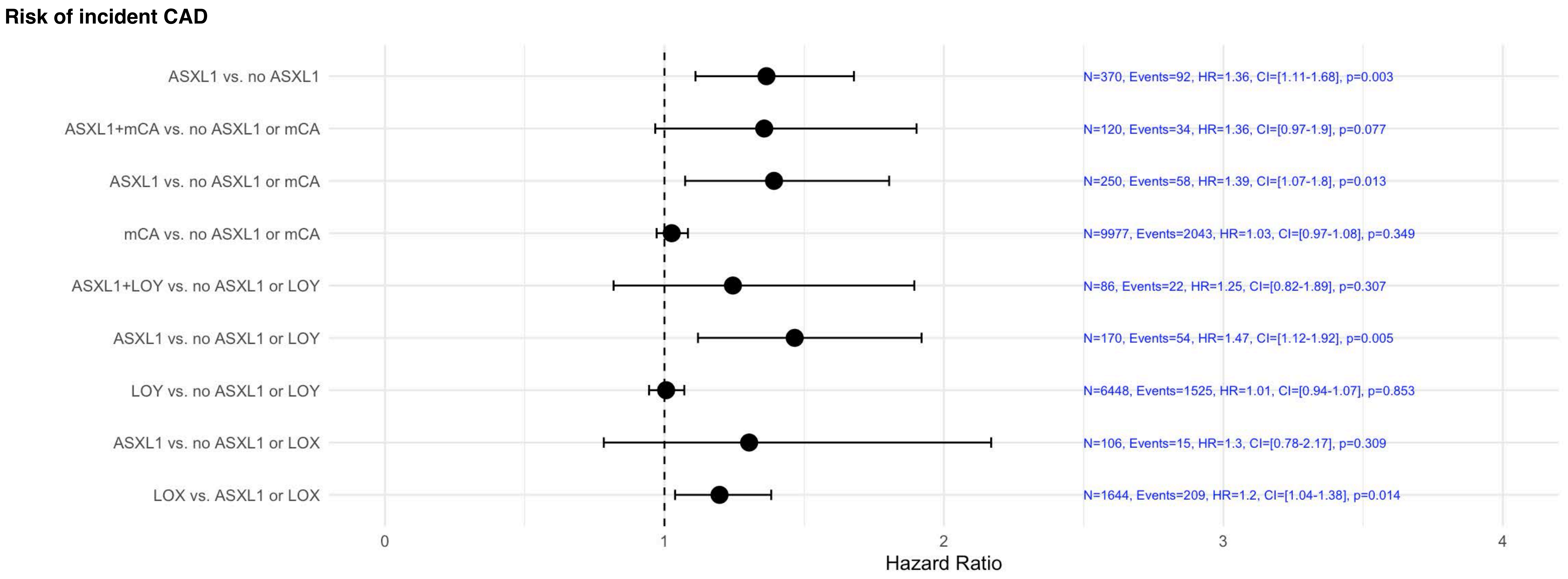
Risk of incident CAD
ASXL1 vs. no ASXL1
ASXL1+mCA vs. no ASXL1 or mCA
ASXL1 vs. no ASXL1 or mCA
mCA vs. no ASXL1 or mCA
ASXL1+LOY vs. no ASXL1 or LOY
ASXL1 vs. no ASXL1 or LOY
LOY vs. no ASXL1 or LOY
ASXL1 vs. no ASXL1 or LOX
LOX vs. ASXL1 or LOX
N=370, Events=92, HR=1.36, CI=[1.11-1.68], p=0.003
N=120, Events=34, HR=1.36, CI=[0.97-1.9], p=0.077
N=250, Events=58, HR=1.39, CI=[1.07-1.8], p=0.013
N=9977, Events=2043, HR=1.03, CI=[0.97-1.08], p=0.349
N=86, Events=22, HR=1.25, CI=[0.82-1.89], p=0.307
N=170, Events=54, HR=1.47, CI=[1.12-1.92], p=0.005
N=6448, Events=1525, HR=1.01, CI=[0.94-1.07], p=0.853
N=106, Events=15, HR=1.3, CI=[0.78-2.17], p=0.309
N=1644, Events=209, HR=1.2, CI=[1.04-1.38], p=0.014
0
1
2
3
4
Hazard Ratio

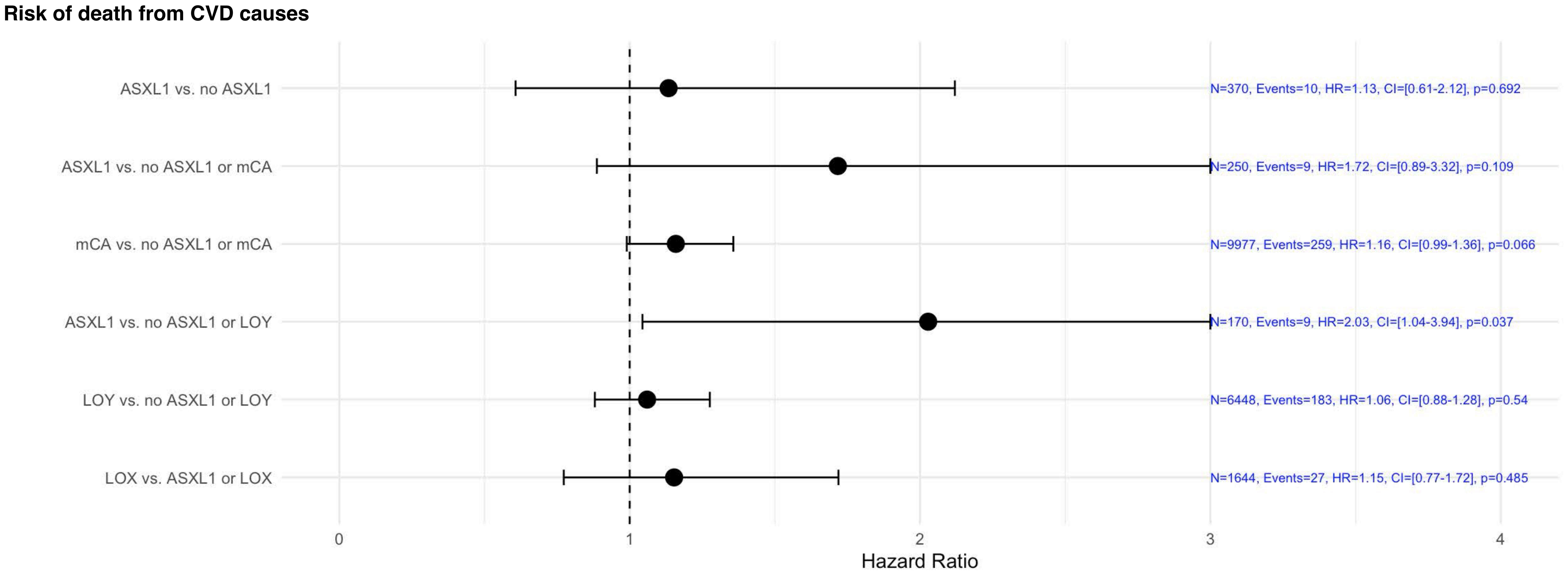
Risk of death from CVD causes
ASXL1 vs. no ASXL1
ASXL1 vs. no ASXL1 or mCA
mCA vs. no ASXL1 or mCA
ASXL1 vs. no ASXL1 or LOY
LOY vs. no ASXL1 or LOY
LOX vs. ASXL1 or LOX
N=370, Events=10, HR=1.13, CI=[0.61-2.12], p=0.692
N=250, Events=9, HR=1.72, CI=[0.89-3.32], p=0.109
N=9977, Events=259, HR=1.16, CI=[0.99-1.36], p=0.066
N=170, Events=9, HR=2.03, CI=[1.04-3.94], p=0.037
N=6448, Events=183, HR=1.06, CI=[0.88-1.28], p=0.54
N=1644, Events=27, HR=1.15, CI=[0.77-1.72], p=0.485
0
1
2
3
4
Hazard Ratio

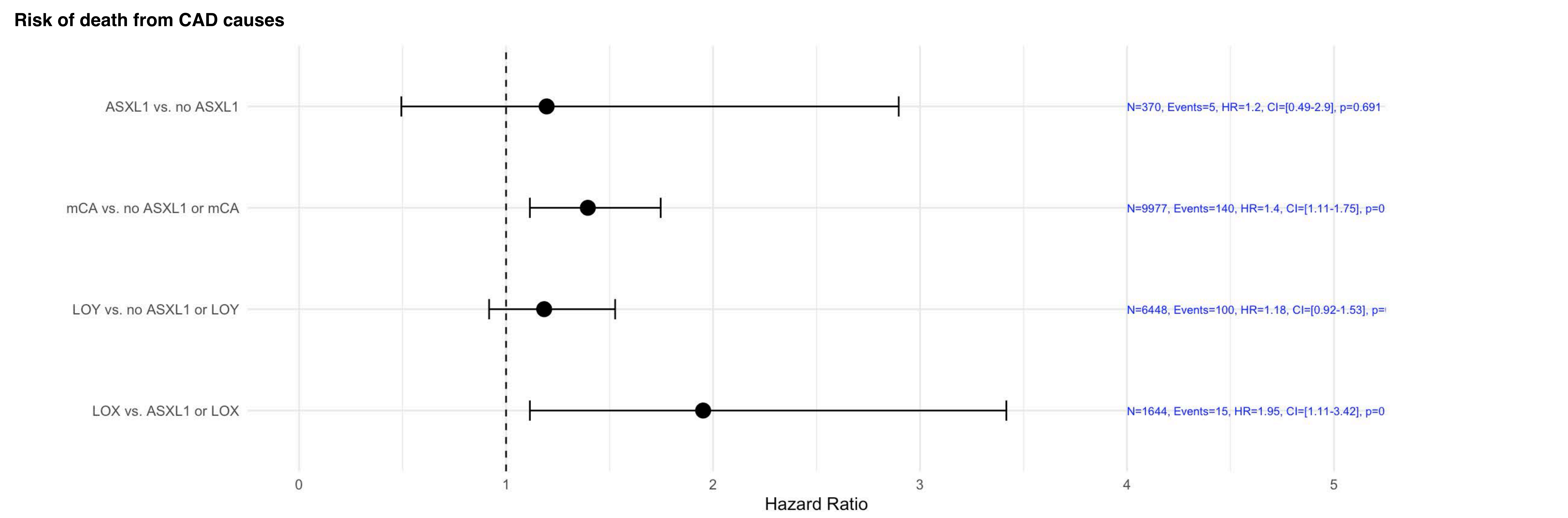
Risk of death from CAD causes
ASXL1 vs. no ASXL1
mCA vs. no ASXL1 or mCA
LOY vs. no ASXL1 or LOY
LOX vs. ASXL1 or LOX
N=370, Events=5, HR=1.2, CI=[0.49-2.9], p=0.691
N=9977, Events=140, HR=1.4, CI=[1.11-1.75], p=0
N=6448, Events=100, HR=1.18, CI=[0.92-1.53], p=
N=1644, Events=15, HR=1.95, CI=[1.11-3.42], p=0
0
1
2
3
4
5
Hazard Ratio

Risk of overall death

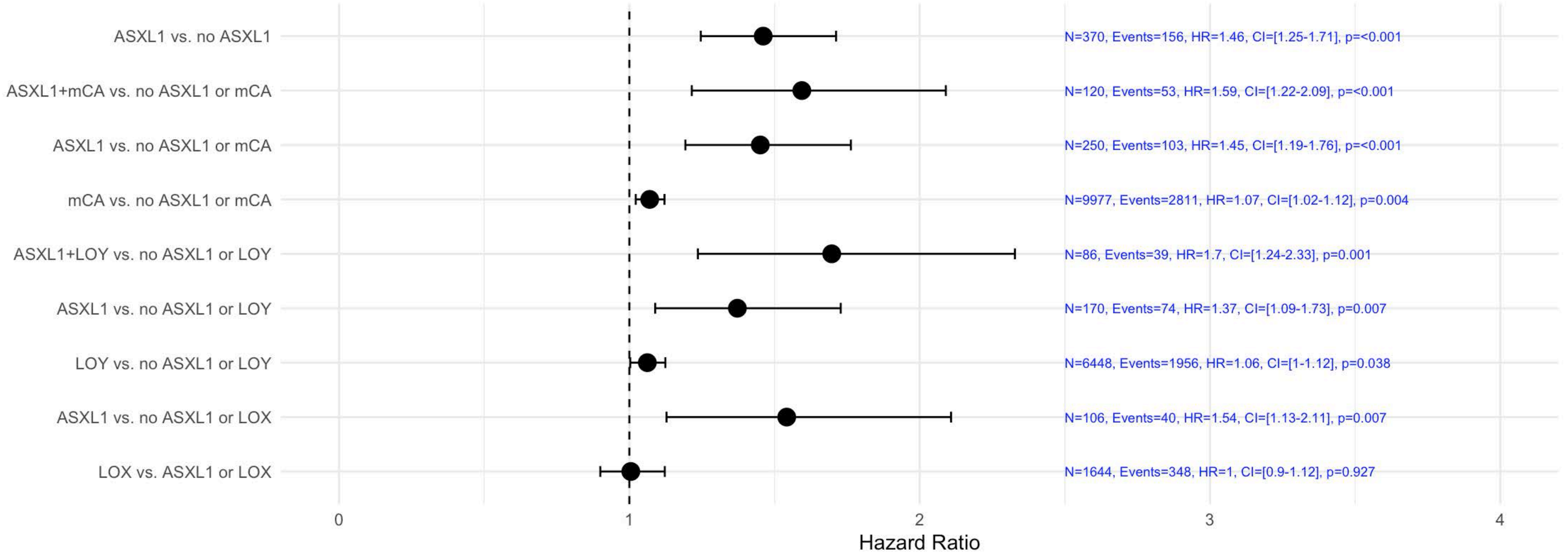

Suppl. Figure 4. Cox regression models assessing the effect of asxl1 co-occurring with mCAs, LOY, or LOX for the risk of incident CVD [1], incident CAD [2], death of CVD causes [3], death of CAD causes [4], and any death [5]. CAD: coronary artery disease, CVD: cardiovascular disease, LOX: mosaic loss of the X chromosome, LOY: mosaic loss of the Y chromosome, mCA: mosaic chromosomal alterations
