# Supplementary material for: Impact of clonal hematopoiesis on cardiovascular outcomes in cancer patients of the UK Biobank
Source: ESMO Open. 2025 Aug 7;10(8):105539. doi: 10.1016/j.esmoop.2025.105539 (PMC12355096; doi:10.1016/j.esmoop.2025.105539)
Supplement: Supplementary Table S1 [file mmc10.docx]

**Supplementary Table S1.** Primary diagnostic codes for selected cancers.

|  | **ICD-9** | **ICD-10** |  |  |  |  |  |
| --- | --- | --- | --- | --- | --- | --- | --- |
| Urinary bladder | 188x | C67x |  |  |  |  |  |
| Larynx | 161.0, 161.1, 161.3, 161.9 | C32x |  |  |  |  |  |
| Prostate | 185 | C61 |  |  |  |  |  |
| Corpus uteri | 182 | C54x |  |  |  |  |  |
| Rectal | 154.1 | C20 |  |  |  |  |  |
| Breast | 174x | C50x |  |  |  |  |  |
| Kidney | 189 | C64 |  |  |  |  |  |
| Melanoma of the skin | 172.x | C43x |  |  |  |  |  |
|  |  |  |  |  |  |  |  |
| Lung and bronchus | 162.x | C34x |  |  |  |  |  |
| All cancer cases were identified using the cancer register ICD-19 and ICD-10 diagnostic codes (data-fields 40013 and 40006) | | | | | | | |
