# Supplementary material for: Impact of clonal hematopoiesis on cardiovascular outcomes in cancer patients of the UK Biobank
Source: ESMO Open. 2025 Aug 7;10(8):105539. doi: 10.1016/j.esmoop.2025.105539 (PMC12355096; doi:10.1016/j.esmoop.2025.105539)
Supplement: Supplementary Table S2 [file mmc11.docx]

**Supplementary Table S2.** Descriptive characteristics of patients diagnosed with cancers susceptible to cardiovascular-related health issues, UK Biobank (n=49,159).

| **Characteristic** | **Overall**  N = 49,159 | **No mCA**  N = 39,002 | **mCA**  N = 10,157 | **OR***^1^* | **95% CI***^1^* | **p-value** |
| --- | --- | --- | --- | --- | --- | --- |
| **Age at bsl, yr** |  |  |  | 1.095 | 1.090, 1.099 | <0.001 |
| Mean (SD) | 60 (7) | 60 (7) | 63 (5) |  |  |  |
| Median (IQR) | 62 (57, 65) | 61 (55, 65) | 64 (61, 67) |  |  |  |
| Range | 40, 71 | 40, 70 | 40, 71 |  |  |  |
| **Sex** |  |  |  |  |  |  |
| Female | 26,623 (54.2%) | 23,930 (61.4%) | 2,693 (26.5%) | Ref. | — | — |
| Male | 22,536 (45.8%) | 15,072 (38.6%) | 7,464 (73.5%) | 3.817 | 3.633, 4.011 | <0.001 |
| **Smoking status** |  |  |  |  |  |  |
| Current smoker | 5,513 (11.3%) | 3,989 (10.3%) | 1,524 (15.1%) | Ref. | — | — |
| Never smoker | 23,968 (49.1%) | 19,957 (51.5%) | 4,011 (39.7%) | 0.548 | 0.509, 0.590 | <0.001 |
| Previous smoker | 19,381 (39.7%) | 14,819 (38.2%) | 4,562 (45.2%) | 0.641 | 0.596, 0.690 | <0.001 |
| Unknown | 297 | 237 | 60 |  |  |  |
| **Chemotherapy** | 11,477 (23.3%) | 9,279 (23.8%) | 2,198 (21.6%) | 1.130 | 1.068, 1.196 | <0.001 |
| **Radiotherapy** | 3,086 (6.3%) | 2,404 (6.2%) | 682 (6.7%) | 0.972 | 0.885, 1.067 | 0.555 |
| **Prevalent CVD** | 11,126 (22.6%) | 8,342 (21.4%) | 2,784 (27.4%) | 1.029 | 0.975, 1.085 | 0.299 |
| **Any CHIP** | 2,701 (5.5%) | 2,006 (5.1%) | 695 (6.8%) | 1.058 | 0.962, 1.162 | 0.246 |
| **Expanded CHIP** | 1,713 (3.5%) | 1,246 (3.2%) | 467 (4.6%) | 1.127 | 1.003, 1.264 | 0.042 |
| ***DNMT3A*** | 1,417 (2.9%) | 1,126 (2.9%) | 291 (2.9%) | 0.873 | 0.759, 1.001 | 0.054 |
| ***TET2*** | 614 (1.2%) | 431 (1.1%) | 183 (1.8%) | 1.237 | 1.025, 1.486 | 0.025 |
| ***ASXL1*** | 374 (0.8% | 253 (0.6%) | 120 (1.2%) | 1.112 | 0.882, 1.397 | 0.364 |
| **Expanded mCA** | 1,946 (4.0%) | - | 1,946 (19.2%) | — | — | — |

CAD: coronary artery disease, CHIP: clonal hematopoiesis of indeterminate potential, CI: confidence interval, CVD: cardiovascular disease, IQR: interquartile range, mCA: mosaic chromosomal alterations, OR: odds ratio, Ref.: referent category, SD: standard deviation

Expanded CHIP means those with variant allele frequency (VAF) ≥10%; expanded mCA means cell fraction >10%
