# Supplementary material for: Impact of clonal hematopoiesis on cardiovascular outcomes in cancer patients of the UK Biobank
Source: ESMO Open. 2025 Aug 7;10(8):105539. doi: 10.1016/j.esmoop.2025.105539 (PMC12355096; doi:10.1016/j.esmoop.2025.105539)
Supplement: Supplementary Table S3 [file mmc12.docx]

**Supplementary Table S3.** Logistic regression analyses assessing the odds of CHIP mutations according to cancer types.

|  | **N** | **OR** | **Lower CI** | **Upper CI** | **P** |
| --- | --- | --- | --- | --- | --- |
| Lung cancer | 4244 | 1.569 | 1.395 | 1.759 | <0.001 |
| Bladder cancer | 1753 | 1.125 | 0.928 | 1.352 | 0.219 |
| Kidney cancer | 1918 | 1.079 | 0.884 | 1.303 | 0.444 |
| Prostate cancer | 13342 | 0.924 | 0.847 | 1.007 | 0.074 |
| Corpus uteri | 2340 | 0.917 | 0.749 | 1.112 | 0.390 |
| Breast cancer | 17365 | 0.891 | 0.818 | 0.971 | 0.008 |
| Larynx cancer | 375 | 0.89 | 0.553 | 1.353 | 0.607 |
| Rectal cancer | 2276 | 0.756 | 0.613 | 0.922 | 0.007 |

*All models adjusted for age and sex (except for prostate, breast, and corpus uteri cancers). CI: confidence interval, CHIP: clonal hematopoiesis of indeterminate potential, OR: odds ratio*
