# Supplementary material for: Impact of clonal hematopoiesis on cardiovascular outcomes in cancer patients of the UK Biobank
Source: ESMO Open. 2025 Aug 7;10(8):105539. doi: 10.1016/j.esmoop.2025.105539 (PMC12355096; doi:10.1016/j.esmoop.2025.105539)
Supplement: Supplementary Table S4 [file mmc13.docx]

**Supplementary Table S4.** Logistic regression analyses assessing the odds of mCAs according to cancer types.

| **Cancer type** | **N** | **Event N** | **OR** | **95% CI** | **P** |
| --- | --- | --- | --- | --- | --- |
| Bladder cancer | 49,159 | 10157 | 1.092 | 0.979, 1.216 | 0.113 |
| Larynx cancer | 49,159 | 10157 | 1.172 | 0.937, 1.461 | 0.161 |
| Corpus uteri | 49,159 | 10157 | 0.847 | 0.731, 0.978 | 0.025 |
| Prostate cancer | 49,159 | 10157 | 2.086 | 1.990, 2.187 | <0.001 |
| Breast cancer | 49,519 | 10157 | 0.33 | 0.311, 0.349 | <0.001 |
| Rectal cancer | 49,159 | 10157 | 0.95 | 0.855, 1.054 | 0.339 |
| Kidney cancer | 49,159 | 10157 | 0.931 | 0.829, 1.043 | 0.219 |
| Lung cancer | 49,159 | 10157 | 1.299 | 1.204, 1.402 | <0.001 |

*All models adjusted for age and sex (except for prostate, breast, and corpus uteri cancers). CI: confidence interval, mCA: mosaic chromosomal alterations, OR: odds ratio*
