# Supplementary material for: Impact of clonal hematopoiesis on cardiovascular outcomes in cancer patients of the UK Biobank
Source: ESMO Open. 2025 Aug 7;10(8):105539. doi: 10.1016/j.esmoop.2025.105539 (PMC12355096; doi:10.1016/j.esmoop.2025.105539)
Supplement: Supplementary Table S5 [file mmc14.docx]

**Supplementary Table S5.** Logistic regression analyses assessing the odds of CHIP mutations in women with breast cancer (n=17,285).

| **Characteristic** | **N** | **Event N** | **OR***^1^* | **95% CI***^1^* | **p-value** |  |
| --- | --- | --- | --- | --- | --- | --- |
| Age at baseline | 17,285 | 802 | 1.08 | 1.064, 1.091 | <0.001 |  |
| Chemotherapy | 17,285 | 802 | 1.13 | 0.961, 1.327 | 0.134 |  |
| Radiotherapy | 17,285 | 802 | 1.01 | 0.709, 1.394 | 0.959 |  |
| Smoking status |  |  |  |  |  |  |
| Current smoker | 1,476 | 67 | — | — |  |  |
| Never smoker | 9,750 | 450 | 0.93 | 0.718, 1.221 | 0.588 |  |
| Previous smoker | 6,059 | 285 | 0.88 | 0.673, 1.168 | 0.365 |  |
| Any mCA | 17,285 | 802 | 1.44 | 1.168, 1.756 | <0.001 |  |
| *Adjusted for age, chemotherapy, radiotherapy, and smoking status | | | | | | |
| 1 CHIP: clonal hematopoiesis of indeterminate potential, CI: confidence interval, OR: odds ratio | | | | | | |
