# Supplementary material for: Impact of clonal hematopoiesis on cardiovascular outcomes in cancer patients of the UK Biobank
Source: ESMO Open. 2025 Aug 7;10(8):105539. doi: 10.1016/j.esmoop.2025.105539 (PMC12355096; doi:10.1016/j.esmoop.2025.105539)
Supplement: Supplementary Table S6 [file mmc15.docx]

**Supplementary Table S6.** Logistic regression analyses assessing the odds of mCAs in women with breast cancer (n=17,285).

| **Characteristic** | **N** | **Event N** | **OR***^1^* | **95% CI***^1^* | **p-value** |
| --- | --- | --- | --- | --- | --- |
| Age at bsl | 17,285 | 1638 | 1.074 | 1.065, 1.084 | <0.001 |
| Chemotherapy | 17,285 | 1638 | 0.923 | 0.818, 1.040 | 0.193 |
| Radiotherapy | 17,285 | 1638 | 0.911 | 0.700, 1.167 | 0.474 |
| Smoking status |  |  |  |  |  |
| Current smoker | 1,476 | 149 | — | — |  |
| Never smoker | 9,750 | 884 | 0.802 | 0.668, 0.969 | 0.020 |
| Previous smoker | 6,059 | 605 | 0.83 | 0.687, 1.009 | 0.057 |
| Any CHIP | 17,285 | 1638 | 1.44 | 1.170, 1.758 | <0.001 |
| *^1^* CI: confidence interval, mCA: mosaic chromosomal alterations, OR: odds ratio | | | | | |
| *Adjusted for age, CHIP, chemotherapy, radiotherapy, and smoking status | | | | | |
