# Supplementary material for: Impact of clonal hematopoiesis on cardiovascular outcomes in cancer patients of the UK Biobank
Source: ESMO Open. 2025 Aug 7;10(8):105539. doi: 10.1016/j.esmoop.2025.105539 (PMC12355096; doi:10.1016/j.esmoop.2025.105539)
Supplement: Supplementary Table S7 [file mmc16.docx]

**Supplementary Table S7.** Logistic regression analyses assessing the odds of CHIP mutations in patients with rectal cancer (n=2,266).

| **Characteristic** | **N** | **Event N** | **OR***^1^* | **95% CI***^1^* | **p-value** |
| --- | --- | --- | --- | --- | --- |
| Age at bsl | 2,266 | 101 | 1.076 | 1.035, 1.121 | <0.001 |
| Sex |  |  |  |  |  |
| Female | 841 | 37 | — | — |  |
| Male | 1,425 | 64 | 0.951 | 0.618, 1.480 | 0.821 |
| Chemotherapy | 2,266 | 101 | 0.614 | 0.366, 0.987 | 0.053 |
| Radiotherapy | 2,266 | 101 | 0.396 | 0.064, 1.287 | 0.201 |
| Smoking Status |  |  |  |  |  |
| Current smoker | 213 | 18 | — | — |  |
| Never smoker | 990 | 39 | 0.403 | 0.225, 0.748 | 0.003 |
| Previous smoker | 1,063 | 44 | 0.389 | 0.220, 0.712 | 0.002 |
| Any mCA | 2,266 | 101 | 0.749 | 0.446, 1.219 | 0.258 |

**Adjusted for age, sex, chemotherapy, radiotherapy, and smoking status
^1^CHIP: clonal hematopoiesis of indeterminate potential, CI: confidence interval, OR: odds ratio*
