# Supplementary material for: Impact of clonal hematopoiesis on cardiovascular outcomes in cancer patients of the UK Biobank
Source: ESMO Open. 2025 Aug 7;10(8):105539. doi: 10.1016/j.esmoop.2025.105539 (PMC12355096; doi:10.1016/j.esmoop.2025.105539)
Supplement: Supplementary Table S8 [file mmc17.docx]

**Supplementary Table S8.** Logistic regression analyses assessing the odds of CHIP mutations in patients with lung cancer (n=4,196).

| **Characteristic** | **N** | **Event N** | **OR***^1^* | **95% CI***^1^* | **p-value** |
| --- | --- | --- | --- | --- | --- |
| Age at baseline | 4,196 | 362 | 1.086 | 1.061, 1.113 | <0.001 |
| Sex |  |  |  |  |  |
| Female | 2,033 | 149 | — | — |  |
| Male | 2,163 | 213 | 1.316 | 1.039, 1.670 | 0.023 |
| Chemotherapy | 4,196 | 362 | 1.201 | 0.959, 1.501 | 0.108 |
| Radiotherapy | 4,196 | 362 | 1.058 | 0.720, 1.511 | 0.766 |
| Smoking Status |  |  |  |  |  |
| Current smoker | 1,604 | 147 | — | — |  |
| Never smoker | 606 | 41 | 0.736 | 0.505, 1.051 | 0.100 |
| Previous smoker | 1,986 | 174 | 0.801 | 0.632, 1.015 | 0.065 |
| Any mCA | 4,196 | 362 | 0.914 | 0.706, 1.177 | 0.488 |

**Adjusted for age, sex, chemotherapy, radiotherapy, and smoking status
^1^CHIP: clonal hematopoiesis of indeterminate potential, CI: confidence interval, OR: odds ratio*
