# Supplementary material for: Impact of clonal hematopoiesis on cardiovascular outcomes in cancer patients of the UK Biobank
Source: ESMO Open. 2025 Aug 7;10(8):105539. doi: 10.1016/j.esmoop.2025.105539 (PMC12355096; doi:10.1016/j.esmoop.2025.105539)
Supplement: Supplementary Table S9 [file mmc18.docx]

**Supplementary Table S9.** Logistic regression analyses assessing the odds of mCAs in patients with lung cancer (n=4,196).

| **Characteristic** | **N** | **Event N** | **OR***^1^* | **95% CI***^1^* | **p-value** |
| --- | --- | --- | --- | --- | --- |
| Age at bsl | 4,196 | 1140 | 1.106 | 1.089, 1.124 | <0.001 |
| Sex |  |  |  |  |  |
| Female | 2,033 | 228 | — | — |  |
| Male | 2,163 | 912 | 5.605 | 4.754, 6.632 | <0.001 |
| Chemotherapy | 4,196 | 1140 | 1.168 | 1.000, 1.364 | 0.05 |
| Radiotherapy | 4,196 | 1140 | 0.807 | 0.616, 1.049 | 0.114 |
| Smoking status |  |  |  |  |  |
| Current smoker | 1,604 | 484 | — | — |  |
| Never smoker | 606 | 100 | 0.558 | 0.429, 0.722 | <0.001 |
| Previous smoker | 1,986 | 556 | 0.693 | 0.589, 0.815 | <0.001 |
| Any CHIP | 4,196 | 1140 | 0.912 | 0.705, 1.175 | 0.48 |

**Adjusted for age, sex, CHIP, chemotherapy, radiotherapy, and smoking status
^1^CHIP: clonal hematopoiesis of indeterminate potential, CI: confidence interval, mCAs: mosaic chromosomal alterations, OR: odds ratio*
