# Supplementary material for: Impact of clonal hematopoiesis on cardiovascular outcomes in cancer patients of the UK Biobank
Source: ESMO Open. 2025 Aug 7;10(8):105539. doi: 10.1016/j.esmoop.2025.105539 (PMC12355096; doi:10.1016/j.esmoop.2025.105539)
Supplement: Supplementary Table S10 [file mmc19.docx]

**Supplementary Table S10.** Multivariable Cox regression models assessing the risk of CHIP on various cardiovascular-related endpoints.

| **Characteristic** | **N** | **Event N** | **HR** | **95% CI** | ***P*-value** |
| --- | --- | --- | --- | --- | --- |
| **Time to incident CVD** | | | | | |
| No CHIP | 46,187 | 26345 | — | — |  |
| Any CHIP | 2,675 | 1754 | 1.074 | 1.023, 1.127 | 0.004 |
| **Time to incident CAD** | | | | | |
| No CHIP | 46,187 | 6549 | — | — |  |
| Any CHIP | 2,675 | 492 | 1.086 | 0.990, 1.190 | 0.079 |
| **Time to CV death** | | | | | |
| No CHIP | 46,187 | 749 | — | — |  |
| Any CHIP | 2,675 | 56 | 1.029 | 0.784, 1.351 | 0.837 |
| **Time to CAD death** | | | | | |
| No CHIP | 46,187 | 343 | — | — |  |
| Any CHIP | 2,675 | 21 | 0.830 | 0.533, 1.291 | 0.407 |
| **Time to any death** | | | | | |
| No CHIP | 46,187 | 9781 | — | — |  |
| Any CHIP | 2,675 | 859 | 1.309 | 1.220, 1.404 | <0.001 |
| CAD: coronary artery disease, CHIP: clonal hematopoiesis of indeterminate potential, CI: confidence interval, CVD: cardiovascular disease, HR: hazard ratio | | | | | |
| *Models adjusted for age at baseline, sex, smoking status, chemotherapy, radiotherapy, prevalent CVD, number of days between date of recruitment and date of cancer diagnosis, and genotyping principal components 1-10* | | | | | |
