# Supplementary material for: Impact of clonal hematopoiesis on cardiovascular outcomes in cancer patients of the UK Biobank
Source: ESMO Open. 2025 Aug 7;10(8):105539. doi: 10.1016/j.esmoop.2025.105539 (PMC12355096; doi:10.1016/j.esmoop.2025.105539)
Supplement: Supplementary Table S11 [file mmc20.docx]

**Supplementary Table S11.** Multivariable Cox regression models assessing the risk expanded CHIP on various cardiovascular-related endpoints.

| **Characteristic** | **N** | **Event N** | **HR** | **95% CI** | ***P*-value** |
| --- | --- | --- | --- | --- | --- |
| **Time to incident CVD** | | | | | |
| VAF<10% | 47,161 | 26938 | — | — |  |
| VAF≥10% | 1,701 | 1161 | 1.133 | 1.068, 1.202 | <0.001 |
| **Time to incident CAD** | | | | | |
| VAF<10% | 47,161 | 6716 | — | — |  |
| VAF≥10% | 1,701 | 325 | 1.103 | 0.986, 1.233 | 0.087 |
| **Time to CV death** | | | | | |
| VAF<10% | 47,161 | 764 | — | — |  |
| VAF≥10% | 1,701 | 41 | 1.181 | 0.862, 1.618 | 0.301 |
| **Time to CAD death** | | | | | |
| VAF<10% | 47,161 | 350 | — | — |  |
| VAF≥10% | 1,701 | 14 | 0.864 | 0.506, 1.476 | 0.592 |
| **Time to any death** | | | | | |
| VAF<10% | 47,161 | 10044 | — | — |  |
| VAF≥10% | 1,701 | 596 | 1.422 | 1.309, 1.545 | <0.001 |
| CAD: coronary artery disease, CHIP: clonal hematopoiesis of indeterminate potential, CI: confidence interval, CVD: cardiovascular disease, HR: hazard ratio, VAF: variant allele frequency | | | | | |
| *Models adjusted for age at baseline, sex, smoking status, chemotherapy, radiotherapy, prevalent CVD, number of days between date of recruitment and date of cancer diagnosis, and genotyping principal components 1-10.*  *Expanded CHIP means mutations with a variant allele frequency (VAF) of ≥10%* | | | | | |
