# Supplementary material for: Impact of clonal hematopoiesis on cardiovascular outcomes in cancer patients of the UK Biobank
Source: ESMO Open. 2025 Aug 7;10(8):105539. doi: 10.1016/j.esmoop.2025.105539 (PMC12355096; doi:10.1016/j.esmoop.2025.105539)
Supplement: Supplementary Table S12 [file mmc21.docx]

**Supplementary Table S12.** Multivariable Cox regression models assessing the risk expanded CHIP and mCA on various cardiovascular-related endpoints.

| **Characteristic** | **N** | **Event N** | **HR** | **95% CI** | ***P*-value** |
| --- | --- | --- | --- | --- | --- |
| **Time to incident CVD** | | | | | |
| No CH or CHIP VAF<10% | 37,527 | 20572 | — | — |  |
| Both | 463 | 333 | 1.084 | 0.972, 1.209 | 0.146 |
| CHIP VAF≥10% only | 1,238 | 828 | 1.152 | 1.074, 1.235 | <0.001 |
| mCA only | 9,634 | 6366 | 0.995 | 0.966, 1.026 | 0.757 |
| **Time to incident CAD** | | | | | |
| No CH or CHIP VAF<10% | 37,527 | 4745 | — | — |  |
| Both | 463 | 106 | 1.103 | 0.909, 1.338 | 0.322 |
| CHIP VAF≥10% only | 1,238 | 219 | 1.117 | 0.975, 1.280 | 0.109 |
| mCA only | 9,634 | 1971 | 1.028 | 0.972, 1.087 | 0.330 |
| **Time to CV death** | | | | | |
| No CH or CHIP VAF<10% | 37,527 | 519 | — | — |  |
| Both | 463 | 15 | 1.381 | 0.824, 2.315 | 0.221 |
| CHIP VAF≥10% only | 1,238 | 26 | 1.161 | 0.782, 1.724 | 0.459 |
| mCA only | 9,634 | 245 | 1.135 | 0.966, 1.333 | 0.124 |
| **Time to CAD death** | | | | | |
| No CH or CHIP VAF<10% | 37,527 | 216 | — | — |  |
| Both | 463 | 7 | 1.434 | 0.672, 3.059 | 0.352 |
| CHIP VAF≥10% only | 1,238 | 7 | 0.732 | 0.344, 1.556 | 0.417 |
| mCA only | 9,634 | 134 | 1.351 | 1.076, 1.698 | 0.010 |
| **Time to any death** | | | | | |
| No CH or CHIP VAF<10% | 37,527 | 7374 | — | — |  |
| Both | 463 | 194 | 1.531 | 1.326, 1.768 | <0.001 |
| CHIP VAF≥10% only | 1,238 | 402 | 1.414 | 1.279, 1.564 | <0.001 |
| mCA only | 9,634 | 2670 | 1.068 | 1.018, 1.119 | 0.007 |
| CAD: coronary artery disease, CH: clonal hematopoiesis, CHIP: clonal hematopoiesis of  indeterminate potential, CI: confidence interval, CVD: cardiovascular disease,  HR: hazard ratio, mCA: mosaic chromosomal alterations, VAF: variant allele frequency | | | | | |
| *Models adjusted for age at baseline, sex, smoking status, chemotherapy,*  *radiotherapy, prevalent CVD, number of days between date of recruitment*  *and date of cancer diagnosis, and genotyping principal components 1-10.*  *Expanded CHIP means clones with VAF≥10%* | | | | | |
