# Supplementary material for: Impact of clonal hematopoiesis on cardiovascular outcomes in cancer patients of the UK Biobank
Source: ESMO Open. 2025 Aug 7;10(8):105539. doi: 10.1016/j.esmoop.2025.105539 (PMC12355096; doi:10.1016/j.esmoop.2025.105539)
Supplement: Supplementary Table S13 [file mmc22.docx]

**Supplementary Table S13.** Multivariable Cox regression models assessing the risk CHIP and expanded mCA on various cardiovascular-related endpoints.

| **Characteristic** | **N** | **Event N** | **HR***^1^* | **95% CI***^1^* | **p-value** |
| --- | --- | --- | --- | --- | --- |
| **Time to incident CVD** | | | | | |
| No CH or cell fraction <10% | 44,419 | 25339 | — | — |  |
| Both | 170 | 110 | 1.138 | 0.943, 1.373 | 0.176 |
| CHIP alone | 2,505 | 1644 | 1.071 | 1.018, 1.126 | 0.008 |
| Expanded mCA | 1,768 | 1006 | 1.020 | 0.957, 1.088 | 0.538 |
| **Time to incident CAD** | | | | | |
| No CH or cell fraction <10% | 44,419 | 6304 | — | — |  |
| Both | 170 | 31 | 1.316 | 0.924, 1.874 | 0.128 |
| CHIP alone | 2,505 | 461 | 1.081 | 0.983, 1.189 | 0.108 |
| Expanded mCA | 1,768 | 245 | 1.227 | 1.077, 1.398 | 0.002 |
| **Time to CVD death** | | | | | |
| No CH or cell fraction <10% | 44,419 | 718 | — | — |  |
| Both | 170 | 2 | 0.579 | 0.144, 2.326 | 0.442 |
| CHIP alone | 2,505 | 54 | 1.067 | 0.808, 1.408 | 0.648 |
| Expanded mCA | 1,768 | 31 | 1.189 | 0.823, 1.718 | 0.357 |
| **Time to CAD death** | | | | | |
| No CH or cell fraction <10% | 44,419 | 328 | — | — |  |
| Both | 170 | 1 | 0.774 | 0.108, 5.525 | 0.798 |
| CHIP alone | 2,505 | 20 | 0.848 | 0.539, 1.333 | 0.474 |
| Expanded mCA | 1,768 | 15 | 1.643 | 0.962, 2.804 | 0.069 |
| **Time to any death** | | | | | |
| No CH or cell fraction <10% | 44,419 | 9398 | — | — |  |
| Both | 170 | 64 | 1.323 | 1.034, 1.693 | 0.026 |
| CHIP alone | 2,505 | 795 | 1.309 | 1.217, 1.408 | <0.001 |
| Expanded mCA | 1,768 | 383 | 1.021 | 0.921, 1.133 | 0.689 |
| CHIP: clonal hematopoiesis of indeterminate potential, CI: confidence interval,  CVD: cardiovascular disease, HR: hazard ratio, mCA: mosaic chromosomal alterations | | | | | |
| *Models adjusted for age at baseline, sex, smoking status, chemotherapy, radiotherapy, prevalent CVD, number of days between date of recruitment and date of cancer diagnosis, and genotyping principal components 1-10.*  *Expanded mCA means alterations with cell fraction >10%.* | | | | | |
