# Supplementary material for: Impact of clonal hematopoiesis on cardiovascular outcomes in cancer patients of the UK Biobank
Source: ESMO Open. 2025 Aug 7;10(8):105539. doi: 10.1016/j.esmoop.2025.105539 (PMC12355096; doi:10.1016/j.esmoop.2025.105539)
Supplement: Supplementary Table S14 [file mmc23.docx]

**Supplementary Table S14.** Multivariable Cox regression models assessing the risk CHIP on various cardiovascular-related endpoint in patients with bladder cancer (n=1,744).

| **Characteristic** | **N** | **Event N** | **HR***^1^* | **95% CI***^1^* | **p-value** | **p-value interaction*** |
| --- | --- | --- | --- | --- | --- | --- |
| Time to CV death | | | | | |  |
| CHIP (any vs. none) | 1,744 | 55 | 0.66 | 0.204, 2.140 | 0.489 | 0.478 |
| Time to CAD death | | | | | |  |
| CHIP (any vs. none) | 1,744 | 26 | - | - | - |  |
| Time to any death | | | | | |  |
| CHIP (any vs. none) | 1,744 | 629 | 1.201 | 0.911, 1.583 | 0.193 | 0.174 |
| Time to incident CVD | | | | | |  |
| CHIP (any vs. none) | 1,744 | 1266 | 0.946 | 0.765, 1.169 | 0.606 | 0.208 |
| Time to incident CAD | | | | | |  |
| CHIP (any vs. none) | 1,744 | 429 | 0.793 | 0.542, 1.160 | 0.232 | 0.056 |

*^1^HR: hazard ratio, CI: confidence interval*

*Models adjusted fo age at baseline, sex, smoking status, chemotherapy, radiotherapy, prevalent CVD, number of days between date of recruitment and date of cancer diagnosis, and genotyping principal components 1-10.*

**CHIP-by-cancer type interaction term P-value in the overall population (n=49,159)*
