# Supplementary material for: Impact of clonal hematopoiesis on cardiovascular outcomes in cancer patients of the UK Biobank
Source: ESMO Open. 2025 Aug 7;10(8):105539. doi: 10.1016/j.esmoop.2025.105539 (PMC12355096; doi:10.1016/j.esmoop.2025.105539)
Supplement: Supplementary Table S15 [file mmc24.docx]

**Supplementary Table S15.** Multivariable Cox regression models assessing the risk CHIP on various cardiovascular-related endpoint in women with breast cancer (n=17,285).

| **Characteristic** | **N** | **Event N** | **HR***^1^* | **95% CI***^1^* | **p-value** | **p-value interaction*** |
| --- | --- | --- | --- | --- | --- | --- |
| Time to CV death | | | | | |  |
| CHIP (any vs. none) | 17,285 | 154 | 0.75 | 0.351, 1.603 | 0.458 | 0.562 |
| Time to CAD death | | | | | |  |
| CHIP (any vs. none) | 17,285 | 43 | 0.734 | 0.177, 3.044 | 0.67 | 0.829 |
| Time to any death | | | | | |  |
| CHIP (any vs. none) | 17,285 | 2377 | 1.299 | 1.106, 1.527 | 0.001 | 0.271 |
| Time to incident CVD | | | | | |  |
| CHIP (any vs. none) | 17,285 | 8211 | 1.078 | 0.979, 1.186 | 0.126 | 0.951 |
| Time to incident CAD | | | | | |  |
| CHIP (any vs. none) | 17,285 | 1347 | 1.304 | 1.056, 1.609 | 0.013 | 0.174 |

*^1^HR: hazard ratio, CI: confidence interval*

*Models adjusted fo age at baseline, smoking status, chemotherapy, radiotherapy, prevalent CVD, number of days between date of recruitment and date of cancer diagnosis, and genotyping principal components 1-10.*

**CHIP-by-cancer type interaction term P-value in the overall population (n=49,159)*
