# Supplementary material for: Impact of clonal hematopoiesis on cardiovascular outcomes in cancer patients of the UK Biobank
Source: ESMO Open. 2025 Aug 7;10(8):105539. doi: 10.1016/j.esmoop.2025.105539 (PMC12355096; doi:10.1016/j.esmoop.2025.105539)
Supplement: Supplementary Table S16 [file mmc25.docx]

**Supplementary Table S16.** Multivariable Cox regression models assessing the risk CHIP on various cardiovascular-related endpoint in women with corpus uteric cancer (n=2,322).

| **Characteristic** | **N** | **Event N** | **HR***^1^* | **95% CI***^1^* | **p-value** | **p-value interaction*** |
| --- | --- | --- | --- | --- | --- | --- |
| Time to CV death | | | | | |  |
| CHIP (any vs. none) | 2,322 | 30 | 1.734 | 0.501, 5.997 | 0.385 | 0.369 |
| Time to CAD death | | | | | |  |
| CHIP (any vs. none) | 2,322 | 7 | - | - | - |  |
| Time to any death | | | | | |  |
| CHIP (any vs. none) | 2,322 | 414 | 1.315 | 0.892, 1.941 | 0.167 | 0.916 |
| Time to incident CVD | | | | | |  |
| CHIP (any vs. none) | 2,322 | 1325 | 1.413 | 1.128, 1.771 | 0.003 | 0.024 |
| Time to incident CAD | | | | | |  |
| CHIP (any vs. none) | 2,322 | 221 | 2.309 | 1.498, 3.559 | <0.001 | 0.001 |

*^1^HR: hazard ratio, CI: confidence interval*

*Models adjusted fo age at baseline, smoking status, chemotherapy, radiotherapy, prevalent CVD, number of days between date of recruitment and date of cancer diagnosis, and genotyping principal components 1-10.*

**CHIP-by-cancer type interaction term P-value in the overall population (n=49,159)*
