# Supplementary material for: Impact of clonal hematopoiesis on cardiovascular outcomes in cancer patients of the UK Biobank
Source: ESMO Open. 2025 Aug 7;10(8):105539. doi: 10.1016/j.esmoop.2025.105539 (PMC12355096; doi:10.1016/j.esmoop.2025.105539)
Supplement: Supplementary Table S17 [file mmc26.docx]

**Supplementary Table S17.** Multivariable Cox regression models assessing the risk CHIP on various cardiovascular-related endpoint in patients with kidney cancer (n=1,908).

| **Characteristic** | **N** | **Event N** | **HR***^1^* | **95% CI***^1^* | **p-value** | **p-value interaction*** |
| --- | --- | --- | --- | --- | --- | --- |
| Time to CV death | | | | | |  |
| CHIP (any vs. none) | 1,908 | 51 | 0.837 | 0.255, 2.745 | 0.769 | 0.569 |
| Time to CAD death | | | | | |  |
| CHIP (any vs. none) | 1,908 | 23 | 0 | 0.000, Inf | 0.997 | 0.987 |
| Time to any death | | | | | |  |
| CHIP (any vs. none) | 1,908 | 633 | 1.223 | 0.912, 1.641 | 0.178 | 0.487 |
| Time to incident CVD | | | | | |  |
| CHIP (any vs. none) | 1,908 | 1420 | 0.869 | 0.694, 1.088 | 0.221 | 0.007 |
| Time to incident CAD | | | | | |  |
| CHIP (any vs. none) | 1,908 | 377 | 0.713 | 0.459, 1.109 | 0.133 | 0.061 |

*^1^HR: hazard ratio, CI: confidence interval*

*Models adjusted fo age at baseline, sex, smoking status, chemotherapy, radiotherapy, prevalent CVD, number of days between date of recruitment and date of cancer diagnosis, and genotyping principal components 1-10.*

**CHIP-by-cancer type interaction term P-value in the overall population (n=49,159)*
