# Supplementary material for: Impact of clonal hematopoiesis on cardiovascular outcomes in cancer patients of the UK Biobank
Source: ESMO Open. 2025 Aug 7;10(8):105539. doi: 10.1016/j.esmoop.2025.105539 (PMC12355096; doi:10.1016/j.esmoop.2025.105539)
Supplement: Supplementary Table S18 [file mmc27.docx]

**Supplementary Table S18.** Multivariable Cox regression models assessing the risk CHIP on various cardiovascular-related endpoint in patients with larynx cancer (n=372).

| **Characteristic** | **N** | **Event N** | **HR***^1^* | **95% CI***^1^* | **p-value** | **p-value interaction*** |
| --- | --- | --- | --- | --- | --- | --- |
| Time to CV death | | | | | |  |
| CHIP (any vs. none) | 372 | 15 | 0 | 0.000, Inf | 0.998 | 0.979 |
| Time to CAD death | | | | | |  |
| CHIP (any vs. none) | 372 | 11 | 0 | 0.000, Inf | >0.999 | 0.987 |
| Time to any death | | | | | |  |
| CHIP (any vs. none) | 372 | 116 | 0.981 | 0.391, 2.459 | 0.967 | 0.800 |
| Time to incident CVD | | | | | |  |
| CHIP (any vs. none) | 372 | 274 | 1.026 | 0.591, 1.781 | 0.927 | 0.893 |
| Time to incident CAD | | | | | |  |
| CHIP (any vs. none) | 372 | 94 | 1.031 | 0.411, 2.589 | 0.948 | 0.721 |

*^1^HR: hazard ratio, CI: confidence interval*

*Models adjusted fo age at baseline, sex, smoking status, chemotherapy, radiotherapy, prevalent CVD, number of days between date of recruitment and date of cancer diagnosis, and genotyping principal components 1-10.*

**CHIP-by-cancer type interaction term P-value in the overall population (n=49,159)*
