# Supplementary material for: Impact of clonal hematopoiesis on cardiovascular outcomes in cancer patients of the UK Biobank
Source: ESMO Open. 2025 Aug 7;10(8):105539. doi: 10.1016/j.esmoop.2025.105539 (PMC12355096; doi:10.1016/j.esmoop.2025.105539)
Supplement: Supplementary Table S19 [file mmc28.docx]

**Supplementary Table S19.** Multivariable Cox regression models assessing the risk CHIP on various cardiovascular-related endpoint in patients with lung cancer (n=4,196).

| **Characteristic** | **N** | **Event N** | **HR***^1^* | **95% CI***^1^* | **p-value** | **p-value interaction*** |
| --- | --- | --- | --- | --- | --- | --- |
| Time to CV death | | | | | |  |
| CHIP (any vs. none) | 4,196 | 109 | 0.872 | 0.452, 1.683 | 0.683 | 0.64 |
| Time to CAD death | | | | | |  |
| CHIP (any vs. none) | 4,196 | 55 | 0.888 | 0.350, 2.254 | 0.802 | 0.908 |
| Time to any death | | | | | |  |
| CHIP (any vs. none) | 4,196 | 3106 | 1.014 | 0.896, 1.148 | 0.823 | 0.001 |
| Time to incident CVD | | | | | |  |
| CHIP (any vs. none) | 4,196 | 3069 | 0.997 | 0.878, 1.132 | 0.962 | 0.041 |
| Time to incident CAD | | | | | |  |
| CHIP (any vs. none) | 4,196 | 928 | 1.003 | 0.806, 1.249 | 0.976 | 0.345 |

*^1^HR: hazard ratio, CI: confidence interval*

*Models adjusted fo age at baseline, sex, smoking status, chemotherapy, radiotherapy, prevalent CVD, number of days between date of recruitment and date of cancer diagnosis, and genotyping principal components 1-10.*

**CHIP-by-cancer type interaction term P-value in the overall population (n=49,159)*
