# Supplementary material for: Impact of clonal hematopoiesis on cardiovascular outcomes in cancer patients of the UK Biobank
Source: ESMO Open. 2025 Aug 7;10(8):105539. doi: 10.1016/j.esmoop.2025.105539 (PMC12355096; doi:10.1016/j.esmoop.2025.105539)
Supplement: Supplementary Table S20 [file mmc29.docx]

**Supplementary Table S20.** Multivariable Cox regression models assessing the risk CHIP on various cardiovascular-related endpoint in patients with melanoma (n=4,920).

| **Characteristic** | **N** | **Event N** | **HR***^1^* | **95% CI***^1^* | **p-value** | **p-value interaction*** |
| --- | --- | --- | --- | --- | --- | --- |
| Time to CV death | | | | | |  |
| CHIP (any vs. none) | 4,920 | 57 | 1.238 | 0.444, 3.450 | 0.684 | 0.927 |
| Time to CAD death | | | | | |  |
| CHIP (any vs. none) | 4,920 | 28 | 1.738 | 0.511, 5.906 | 0.376 | 0.240 |
| Time to any death | | | | | |  |
| CHIP (any vs. none) | 4,920 | 582 | 1.025 | 0.755, 1.391 | 0.874 | 0.800 |
| Time to incident CVD | | | | | |  |
| CHIP (any vs. none) | 4,920 | 2410 | 1.061 | 0.895, 1.258 | 0.495 | 0.821 |
| Time to incident CAD | | | | | |  |
| CHIP (any vs. none) | 4,920 | 597 | 1.09 | 0.785, 1.512 | 0.608 | 0.997 |

*^1^HR: hazard ratio, CI: confidence interval*

*Models adjusted fo age at baseline, sex, smoking status, chemotherapy, radiotherapy, prevalent CVD, number of days between date of recruitment and date of cancer diagnosis, and genotyping principal components 1-10.*

**CHIP-by-cancer type interaction term P-value in the overall population (n=49,159)*
