# Supplementary material for: Impact of clonal hematopoiesis on cardiovascular outcomes in cancer patients of the UK Biobank
Source: ESMO Open. 2025 Aug 7;10(8):105539. doi: 10.1016/j.esmoop.2025.105539 (PMC12355096; doi:10.1016/j.esmoop.2025.105539)
Supplement: Supplementary Table S21 [file mmc30.docx]

**Supplementary Table S21.** Multivariable Cox regression models assessing the risk CHIP on various cardiovascular-related endpoint in men with prostate cancer (n=13,342).

| **Characteristic** | **N** | **Event N** | **HR***^1^* | **95% CI***^1^* | **p-value** | **p-value interaction*** |
| --- | --- | --- | --- | --- | --- | --- |
| Time to CV death | | | | | |  |
| CHIP (any vs. none) | 13,342 | 270 | 1.006 | 0.630, 1.606 | 0.981 | 0.695 |
| Time to CAD death | | | | | |  |
| CHIP (any vs. none) | 13,342 | 143 | 0.904 | 0.459, 1.781 | 0.771 | 0.720 |
| Time to any death | | | | | |  |
| CHIP (any vs. none) | 13,342 | 2176 | 1.117 | 0.957, 1.304 | 0.161 | 0.842 |
| Time to incident CVD | | | | | |  |
| CHIP (any vs. none) | 13,342 | 8462 | 1.076 | 0.985, 1.175 | 0.103 | 0.587 |
| Time to incident CAD | | | | | |  |
| CHIP (any vs. none) | 13,342 | 2589 | 1.063 | 0.912, 1.240 | 0.435 | 0.864 |

*^1^HR: hazard ratio, CI: confidence interval*

*Models adjusted fo age at baseline, smoking status, chemotherapy, radiotherapy, prevalent CVD, number of days between date of recruitment and date of cancer diagnosis, and genotyping principal components 1-10.*

**CHIP-by-cancer type interaction term P-value in the overall population (n=49,159)*
