# Supplementary material for: Impact of clonal hematopoiesis on cardiovascular outcomes in cancer patients of the UK Biobank
Source: ESMO Open. 2025 Aug 7;10(8):105539. doi: 10.1016/j.esmoop.2025.105539 (PMC12355096; doi:10.1016/j.esmoop.2025.105539)
Supplement: Supplementary Table S22 [file mmc31.docx]

**Supplementary Table S22.** Multivariable Cox regression models assessing the risk CHIP on various cardiovascular-related endpoint in men with rectal cancer (n=2,266).

| **Characteristic** | **N** | **Event N** | **HR***^1^* | **95% CI***^1^* | **p-value** | **p-value interaction*** |
| --- | --- | --- | --- | --- | --- | --- |
| Time to CV death | | | | | |  |
| CHIP (any vs. none) | 2,266 | 38 | 0.998 | 0.236, 4.223 | 0.998 | 0.835 |
| Time to CAD death | | | | | |  |
| CHIP (any vs. none) | 2,266 | 23 | - | - | - |  |
| Time to any death | | | | | |  |
| CHIP (any vs. none) | 2,266 | 636 | 1.345 | 0.961, 1.882 | 0.084 | 0.744 |
| Time to incident CVD | | | | | |  |
| CHIP (any vs. none) | 2,266 | 1475 | 1.09 | 0.860, 1.382 | 0.474 | 0.776 |
| Time to incident CAD | | | | | |  |
| CHIP (any vs. none) | 2,266 | 388 | 0.773 | 0.473, 1.264 | 0.305 | 0.116 |

*^1^HR: hazard ratio, CI: confidence interval*

*Models adjusted fo age at baseline, sex, smoking status, chemotherapy, radiotherapy, prevalent CVD, number of days between date of recruitment and date of cancer diagnosis, and genotyping principal components 1-10.*

**CHIP-by-cancer type interaction term P-value in the overall population (n=49,159)*
